# Mad1’s ability to interact with Mad2 is essential to regulate and monitor meiotic synapsis in *C*. *elegans*

**DOI:** 10.1371/journal.pgen.1009598

**Published:** 2021-11-11

**Authors:** Alice Devigne, Needhi Bhalla

**Affiliations:** Department of Molecular, Cell and Developmental Biology, University of California, Santa Cruz, Santa Cruz, California, United States of America; University of California, Davis, UNITED STATES

## Abstract

Meiotic homolog synapsis is essential to ensure accurate segregation of chromosomes during meiosis. In *C*. *elegans*, proper regulation of synapsis and a checkpoint that monitors synapsis relies on the spindle checkpoint components, Mad1 and Mad2, and Pairing Centers (PCs), cis-acting loci that interact with the nuclear envelope to mobilize chromosomes within the nucleus. Here, we test what specific functions of Mad1 and Mad2 are required to regulate and monitor synapsis. We find that a mutation that prevents Mad1’s localization to the nuclear periphery abolishes the synapsis checkpoint but has no effect on Mad2’s localization to the nuclear periphery or synapsis. By contrast, a mutation that prevents Mad1’s interaction with Mad2 abolishes the synapsis checkpoint, delays synapsis and fails to localize Mad2 to the nuclear periphery. These data indicate that Mad1’s primary role in regulating synapsis is through control of Mad2 and that Mad2 can bind other factors at the nuclear periphery. We also tested whether Mad2’s ability to adopt a specific conformation associated with its activity during spindle checkpoint function is required for its role in meiosis. A mutation that prevents Mad2 from adopting its active conformer fails to localize to the nuclear periphery, abolishes the synapsis checkpoint and exhibits substantial defects in meiotic synapsis. Thus, Mad2, and its regulation by Mad1, is an important regulator of meiotic synapsis in *C*. *elegans*.

## Author summary

Sexual reproduction relies on production of gametes, such as eggs and sperm, which are produced during meiosis. During this specialized cell division, chromosomes replicate, pair with their homologs, undergo synapsis and finally undergo recombination, all of which are required for correct meiotic chromosome segregation. Here, we show that the spindle checkpoint effector, Mad2, and its regulation by Mad1, plays an important role in regulating meiotic synapsis.

## Introduction

Meiosis is a specialized biological process during which cells undergo a single round of DNA replication followed by two successive rounds of cell division. This process produces haploid gametes from diploid organisms. Diploidy is restored during sexual reproduction by the fusion of gametes, such as eggs and sperm, during fertilization, producing embryos. If chromosomes missegregate during meiosis, gametes and, upon their fertilization, embryos, will have the wrong number of chromosomes, also called aneuploidy. Aneuploidy during meiosis is frequently associated with miscarriages, infertility, and birth defects such as Down syndrome.

To ensure that chromosome segregation occurs normally during meiosis, critical events in meiotic prophase are tightly coordinated, monitored and regulated. Briefly, after replication, chromosomes pair with their homologs. Homologous interactions are stabilized by the assembly of a proteinaceous structure, the synaptonemal complex (SC) during a process called synapsis. Synapsis is a prerequisite for crossover recombination to generate linkages, or chiasmata, between homologs. These events are essential to direct proper meiotic chromosome segregation in which homologs and sister chromatids are separated during meiosis I and meiosis II respectively.

Because of their importance, multiple cell cycle checkpoints ensure the normal progression of synapsis and recombination, delay the cell cycle to correct errors and promote the removal of persistent abnormal cells [1]. One such checkpoint response, the synapsis checkpoint, triggers apoptosis to eliminate nuclei with unsynapsed chromosomes in *Caenorhabditis elegans* [2]. This checkpoint relies on Pairing Centers (PCs), cis acting sites at one end of each chromosome that promote pairing and synapsis [2,3]. PCs play an important role, anchoring chromosome ends at the nuclear envelope to enable interaction with the SUN-1/ZYG-12 complex that spans the nuclear envelope; this interaction enables PCs to access the microtubule network in the cytoplasm [4–6], allowing chromosomes to become mobile within the nucleus. This mobilization is a conserved feature of meiotic prophase and essential for pairing and synapsis [7] but the specific role of this mobility is unclear.

The spindle assembly checkpoint (SAC) monitors whether chromosomes are properly attached to the spindle during both mitotic and meiotic chromosome segregation [8]. We recently showed that mitotic spindle assembly checkpoint (SAC) components Mad1, Mad2 and Bub3 are required to negatively regulate synapsis and promote the synapsis checkpoint response in *C*. *elegans* [9]. The genes that encode the *C*. *elegans* orthologs of Mad1 and Mad2 are *mdf-1* and *mdf-2*, respectively. For clarity’s sake, we will refer to these genes as *mdf-1*^*mad-1*^ and *mdf-2*^*mad-2*^ and their respective proteins as MDF-1^MAD-1^ and MDF-2^MAD-2^. MDF-1^MAD-1^ and MDF-2^MAD-2^ localize to the nuclear envelope and interact with SUN-1, leading us to propose that these proteins may regulate and monitor synapsis through the ability of PCs to interact with and move at the nuclear envelope [9].

Here we test aspects of this model by investigating what functional aspects of SAC components are required for an efficient synapsis checkpoint. We show the N-terminal portion of MDF-1^MAD-1^, required for the localization of the protein to the nuclear periphery [10], is also required for the synapsis checkpoint. However, unlike other mutant alleles of *mdf-1*^*mad-1*^, this inability to localize to the nuclear envelope does not affect MDF-2^MAD-2^ localization or synapsis. In contrast, a mutation that affects MDF-1^MAD-1^ ‘s interaction with MDF-2^MAD-2^ is crucial for MDF-2^MAD-2^’s localization at the nuclear envelope, timely synapsis and a functional checkpoint. Finally, we demonstrate that the closed conformation of MDF-2^MAD-2^ is required to regulate and monitor synapsis. Thus, MDF-2^MAD-2^, and its regulation by MDF-1^MAD-1^, is an important regulator of meiotic synapsis in *C*. *elegans*.

## Results

### MDF-1^MAD-1^ ‘s localization to nuclear envelope is required for the synapsis checkpoint but not to regulate synapsis

**Fig 1:**
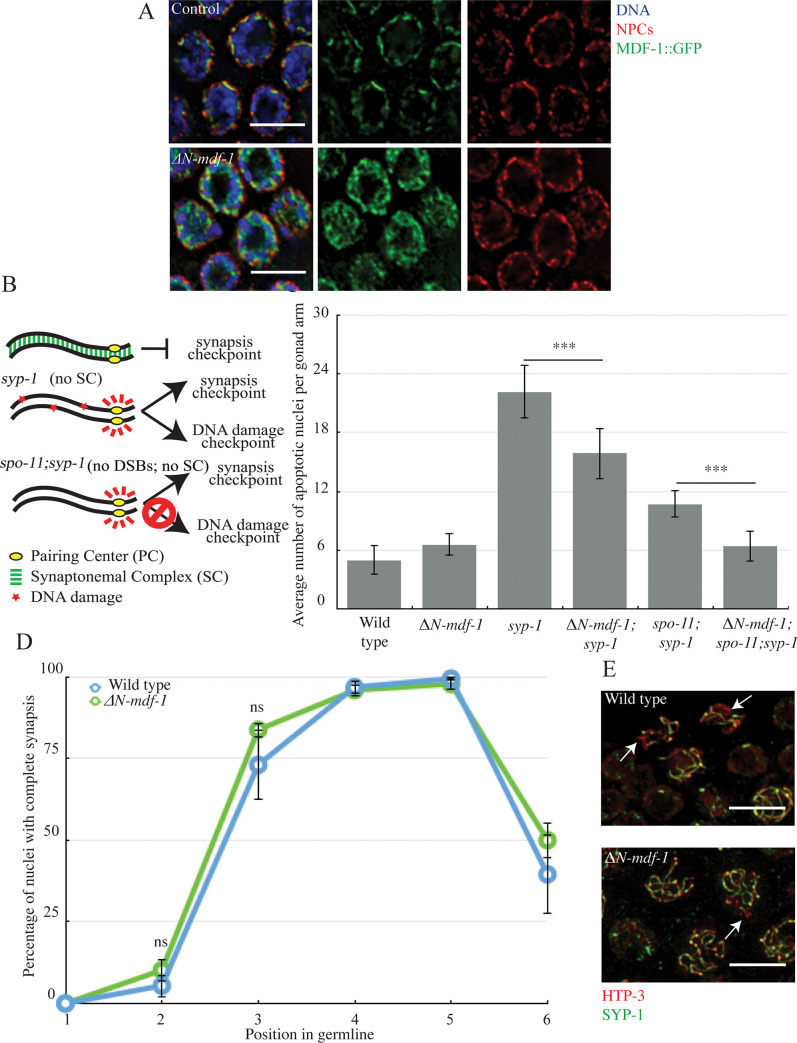
MDF-1^MAD-1^’s localization to the nuclear envelope is required for the synapsis checkpoint but not to regulate synapsis. A. ΔN-MDF-1^MAD-1^ (green) localizes diffusely in the cytoplasm of meiotic nuclei and does not co-localize with NPCs (red). Images are partial projections of meiotic nuclei stained to visualize DNA (blue). Bar: 5 μm. B. A cartoon of meiotic checkpoints in *C*. *elegans*. C. *ΔN-mdf-1*^*mad-1*^ reduces germline apoptosis in *syp-1* and *spo-11;syp-1* mutants. A *** indicates a p value < 0.0001. D. synapsis is unaffected in *ΔN-mdf-1*^*mad-1*^ mutants. ns indicates not significant. E. Images of nuclei during synapsis initiation in wild-type worms and *ΔN-mdf-1*^*mad-1*^ mutants stained to visualize SYP-1 and HTP-3. Arrows indicates unsynapsed chromosomes. Bar: 5 μm.

We previously showed that MDF-1^MAD-1^ localizes to the nuclear periphery during meiotic prophase [9]. Therefore, we tested whether this localization was required for monitoring and regulating synapsis (ΔN-MDF-1^MAD-1^ in S1 Fig). Amino acids 151 to 320 are required for MDF-1^MAD-1^’s interaction with the nuclear pore component Tpr (NPP-21 in *C*. *elegans*) and its localization to the nuclear periphery in mitotic germline cells [10]. Deletion of this region also abolished localization of MDF-1^MAD-1^ at the nuclear periphery of meiotic germline nuclei, as visualized by immunofluorescent staining against nuclear pore complexes (NPCs in Fig 1A). In contrast to control animals with wild-type MDF-1^MAD-1^, ΔN-MDF-1^MAD-1^ adopted a diffuse localization inside nuclei, was excluded from the center of the nucleus where the nucleolus resides and occupied area devoid of DNA (Fig 1A). We performed a line intensity analysis on projections of individual nuclei, as well as a colocalization analysis that plotted the two intensities on a pixel by pixel basis (S2A and S2B Fig), to verify that ΔN-MDF-1^MAD-1^ no longer colocalized with NPCs. In addition, we also stained meiotic nuclei with antibodies against the nuclear envelope protein SUN-1 to verify that ΔN-MDF-1^MAD-1^ was absent from the nuclear envelope (S2C Fig).

Next, we tested what effect this deletion had on the synapsis checkpoint [9]. In *C*. *elegans*, the SC is composed of a family of proteins, one of which is SYP-1. *syp-1* mutants do not load SC between homologs, producing unsynapsed chromosomes [11]. In response to this abnormality, both the synapsis and DNA damage checkpoints are activated, resulting in very high levels of germline apoptosis (Fig 1B and 1C) [2]. When we introduced the Δ*N-mdf-1*^*mad-1*^ deletion into the *syp-1* mutant background, the double mutant exhibited an intermediate level of germline apoptosis, indicating that the ability of MDF-1^MAD-1^ to interact with Tpr and localize to the nuclear periphery is required for either the synapsis or DNA damage checkpoint (Fig 1C). To determine which checkpoint is affected by the loss of the N terminus of MDF-1^MAD-1^, we abolished the DNA damage checkpoint by using the *spo-11;syp-1* mutant background. SPO-11 generates double-strand breaks to initiate meiotic recombination [12]; therefore, in this background only the synapsis checkpoint is activated (Fig 1B) [2]. When we generated the Δ*N-mdf-1*^*mad-1*^*;spo-11;syp-1* triple mutants, we observed levels of apoptosis similar to wild-type animals, indicating the N terminus of MDF-1^MAD-1^ is required for the synapsis checkpoint (Fig 1C).

We previously showed that in some spindle checkpoint mutants, a role in the synapsis checkpoint is coupled to a role in regulating synapsis [9]. To determine whether this is also true for Δ*N-mdf-1*^*mad-*^ deletion mutants, we assessed synapsis progression by staining for two SC proteins. We stained for HTP-3, an axial element that is loaded between sister chromatids before synapsis [3] and for SYP-1 [11]. When we overlay HTP-3 and SYP-1 staining signals, stretches of HTP-3 without SYP-1 indicates the presence of unsynapsed chromosomes (arrows in Fig 1E) while colocalization of HTP-3 and SYP-1 indicates complete synapsis (Fig 1E). In *C*. *elegans*, meiotic nuclei in the germline are organized in a spatiotemporal gradient. Therefore, we divided germlines into six equivalent zones and calculated the percentage per zone of nuclei exhibiting complete synapsis to assay the progression of synapsis (Fig 1D). When we performed this analysis, Δ*N-mdf-1*^*mad-1*^ deletion mutants resembled wild-type germlines (Fig 1D), demonstrating that while the localization of MDF-1^MAD-1^ to the nuclear envelope is required to monitor synapsis (Fig 1C), it is not required to regulate synapsis (Fig 1D). This is in contrast to other mutations in *mdf-1*^*mad-1*^ that both regulate and monitor synapsis [9].

PCH-2 is a highly-conserved AAA+ ATPase that is required for the synapsis checkpoint [2] and coordinates the events of pairing, synapsis and recombination to ensure their fidelity [13,14]. Since Δ*N-mdf-1*^*mad-1*^ deletion mutants abrogated the synapsis checkpoint but did not perturb synapsis, we tested whether PCH-2 localization was affected in this background. In control germlines, PCH-2 localizes to meiotic chromosomes when homologous chromosomes initiate synapsis in the region called the transition zone (TZ) and remains on chromosomes during early and mid-pachytene. PCH-2 is removed from chromosomes when homologs lose the capacity for crossover formation in late pachytene (LP) [13]. Despite the defect in synapsis checkpoint function, PCH-2 localized normally in Δ*N-mdf-1*^*mad-1*^ deletion mutants (S3A Fig), loading on to meiotic chromosomes in the transition zone and was removed in late pachytene (S3B Fig), indicating that the defect in checkpoint function was not the consequence of mislocalization of PCH-2.

### The N terminus of MDF-1^MAD-1^ is not required for MDF-2^MAD-2^’s localization to the nuclear envelope in meiotic germline nuclei

**Fig 2:**
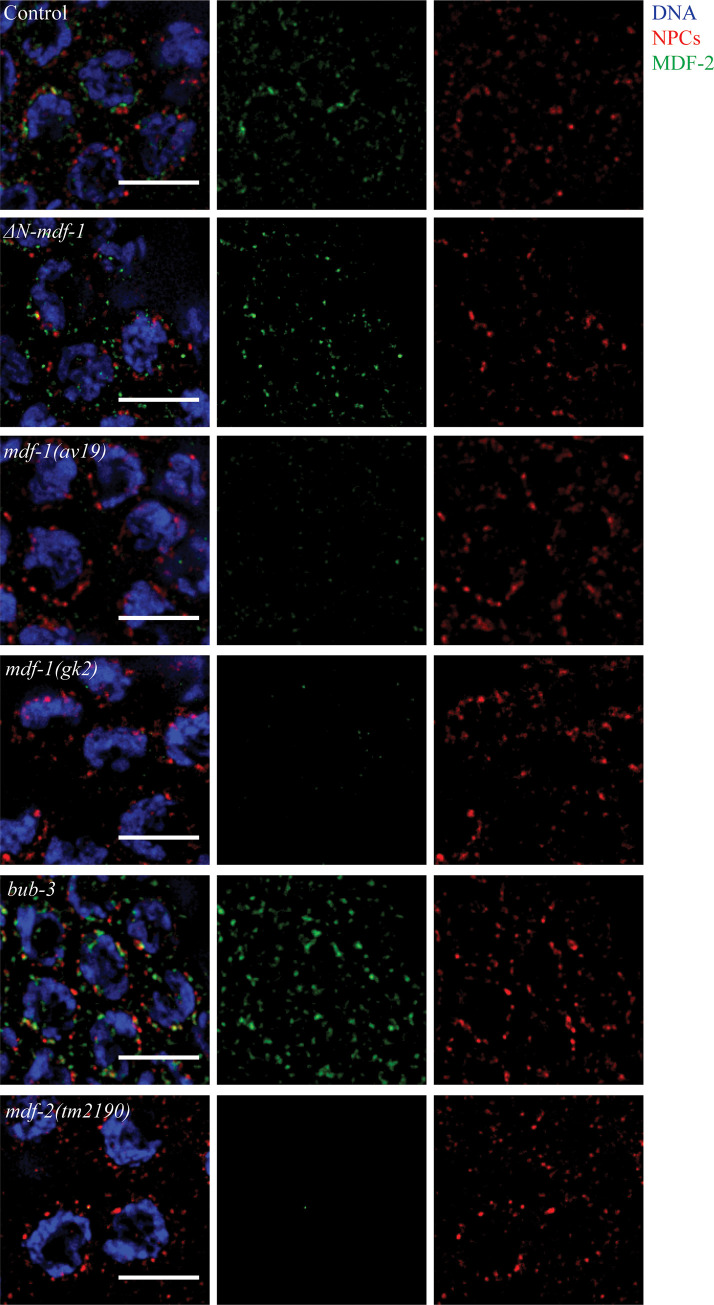
The N-terminus of MDF-1^MAD-1^ is not required for MDF-2^MAD-2^’s localization to the nuclear envelop in meiotic germline nuclei. MDF-2^MAD-2^ (green) co-localizes with NPCs (red) in *ΔN-mdf-1*^*mad-1*^ and *bub-3* mutants but is not detected in *mdf-1*^*mad-1*^*(av19)*, *mdf-1*^*mad-1*^*(gk2)* and *mdf-2*^*mad-2*^*(tm2190)* mutants. Images are partial projections of meiotic nuclei stained to visualize DNA (blue). Bar: 5 μm.

Since Δ*N-mdf-1*^*mad-1*^ mutants did not affect synapsis (Fig 1D), unlike other *mdf-1*^*mad-1*^ mutants we had characterized [9], we tested whether Δ*N-mdf-1*^*mad-1*^ deletion mutants affect the localization of another protein required for the synapsis checkpoint, MDF-2^MAD-2^. MDF-2^MAD-2^ adopts the same localization as MDF-1^MAD-1^ in meiotic germline nuclei: the protein is targeted to the nuclear periphery in a punctate pattern [9]. We performed immunostaining using antibodies against the nuclear envelope protein nuclear pore complexes and MDF-2^MAD-2^ and observed that, in contrast to a mutation that abolishes MDF-1^MAD-1^’s checkpoint function (*mdf-1*^*mad-1*^*[av19]*) [15] and a null mutation in *mdf-1*^*mad-1*^ (*mdf-1*^*mad-1*^*[gk2])*) [16], MDF-2^MAD-2^ localization to the nuclear periphery was unaffected by the MDF-1^MAD-1^’s diffuse staining in Δ*N-mdf-1*^*mad-1*^ deletion mutants (Fig 2). Altogether, these results indicate that while MDF-1^MAD-1^ is required for MDF-2^MAD-2^’s localization in meiotic nuclei, MDF-1^MAD-1^‘s enrichment at the nuclear envelope is not. As a control, we performed immunofluorescence against MDF-2^MAD-2^ in *mdf-2*^*mad-2*^ null mutants (Fig 2). We also verified that MDF-2^MAD-2^ localization was unaffected in *bub-3* mutants (Fig 2), having established a role for this gene in regulating and monitoring synapsis [9].

### MDF-1^MAD-1^’s interaction with BUB-1 is not required to monitor or regulate synapsis

We had previously hypothesized that MDF-1^MAD-1^’s localization to the nuclear periphery in meiotic germline nuclei suggested an interaction with PCs, cis-acting chromosomal regions essential for pairing, synapsis and synapsis checkpoint function [9]. In this way, we compared unsynapsed PCs to unattached kinetochores, which recruit Mad1 and Mad2 to initiate spindle assemble checkpoint signaling [8]. To further explore this connection, we took advantage of a mutation in Mad1 that prevents its localization to unattached kinetochores [17].

**Fig 3:**
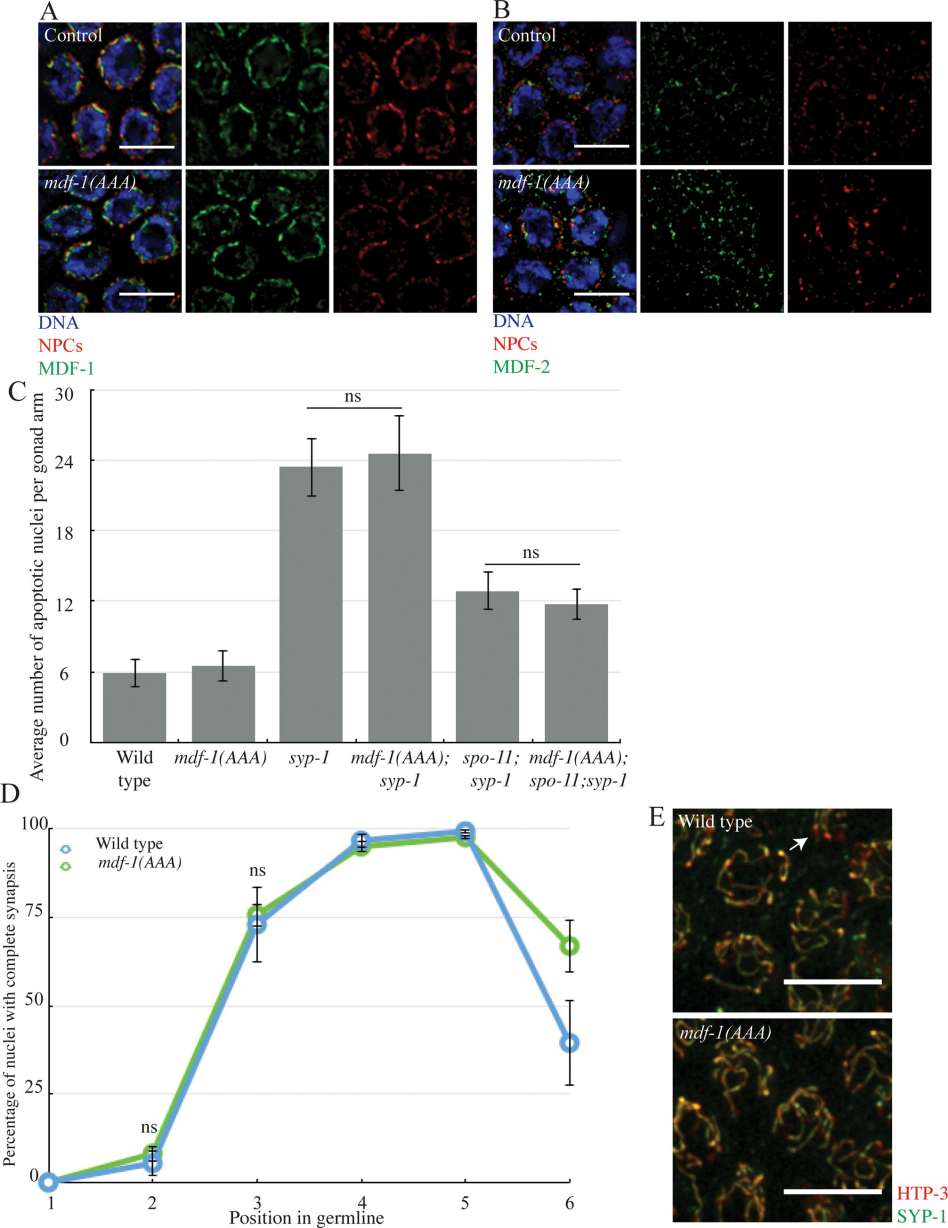
MDF-1^MAD-1^’s interaction with BUB-1 is not required to monitor or regulate synapsis. A. MDF-1^MAD-1^ (green) co-localizes with NPCs (red) in *mdf-1*^*mad-1*^*(AAA)* mutants. B. MDF-2^MAD-2^ (green) co-localizes with NPCs (red) in *mdf-1*^*mad-1*^*(AAA)* mutants. Images are partial projections of meiotic nuclei stained to visualize DNA (blue). Bar: 5 μm. C. The synapsis checkpoint and the DNA damage checkpoint are unperturbed in *mdf-1*^*mad-1*^*(AAA)* mutants. D. Synapsis is unaffected in *mdf-1*^*mad-1*^*(AAA)* mutants. ns indicates not significant. E. Images of nuclei during synapsis initiation in wild-type and *mdf-1*^*mad-1*^*(AAA)* mutants stained to visualize SYP-1 and HTP-3. Arrow indicates unsynapsed chromosomes. Bar: 5 μm.

MDF-1^MAD-1^ is recruited to unattached kinetochores through its interaction with BUB-1, a conserved kinase that is essential for chromosome segregation and spindle checkpoint function [17]. We used a mutant version of MDF-1^MAD-1^, *mdf-1*^*mad-1*^*(E419A*, *R420A*, *D423A)* (S1 Fig) that abolishes its binding to BUB-1, its localization to unattached kinetochores and its function in the spindle checkpoint [17]. We will refer to this allele as *mdf-1*^*mad-1*^*(AAA)*. We tested if MDF-1^MAD-1^’s ability to bind BUB-1 is also required for MDF-1^MAD-1^ localization, checkpoint function and regulation of synapsis in meiosis. We stained fixed germlines with antibodies against SUN-1 and MDF-1^MAD-1^ and observed a localization comparable to wild-type MDF-1^MAD-1^ (Fig 3A). In this mutant, MDF-2^MAD-2^ localization is also unaffected (Fig 3B). Therefore, the region of MDF-1^MAD-1^ that is required to bind BUB-1 and localize to unattached kinetochores is not required for its localization to the nuclear periphery.

Next, we tested whether this motif was required to regulate and monitors synapsis. We generated the double and triple mutants *mdf-1*^*mad-1*^*(AAA)*;*syp-1* and *mdf-1*^*mad-1*^*(AAA);spo-11;syp-1*. When we assayed apoptosis, *mdf-1*^*mad-1*^*(AAA)*;*syp-1* mutants were indistinguishable from *syp-1* single mutants. Similarly, *mdf-1*^*mad-1*^*(AAA);spo-11;syp-1* mutants were indistinguishable from *spo-11;syp-1* mutants (Fig 3C). These results indicate that neither the synapsis or DNA damage checkpoint are affected in the *mdf-1*^*mad-1*^*(AAA)* mutants. When we assayed the progression of synapsis, synapsis in *mdf-1*^*mad-1*^*(AAA)* mutants resembled synapsis in wild-type animals (Fig 3D). Thus, the motif required for MDF-1^MAD-1^’s ability to interact with BUB-1 is not required for the synapsis checkpoint and does not regulate synapsis (Fig 3C and 3D). Consistent with the dispensability of this motif for meiosis, we localized BUB-1 in control, *mdf-1*^*mad-1*^*(AAA)* mutants and other checkpoint deficient strains (Δ*N-mdf-1*^*mad-1*^, *mdf-1*^*mad-1*^(A), and *mdf-2*^*mad-2*^*-open*) identified in this paper (see below), and found that BUB-1 was found outside germline nuclei and unaffected by whether strains had a functional synapsis checkpoint or not (S4 Fig).

### MDF-1^MAD-1^’s ability to interact with MDF-2^MAD-2^ is required to regulate and monitor synapsis

**Fig 4:**
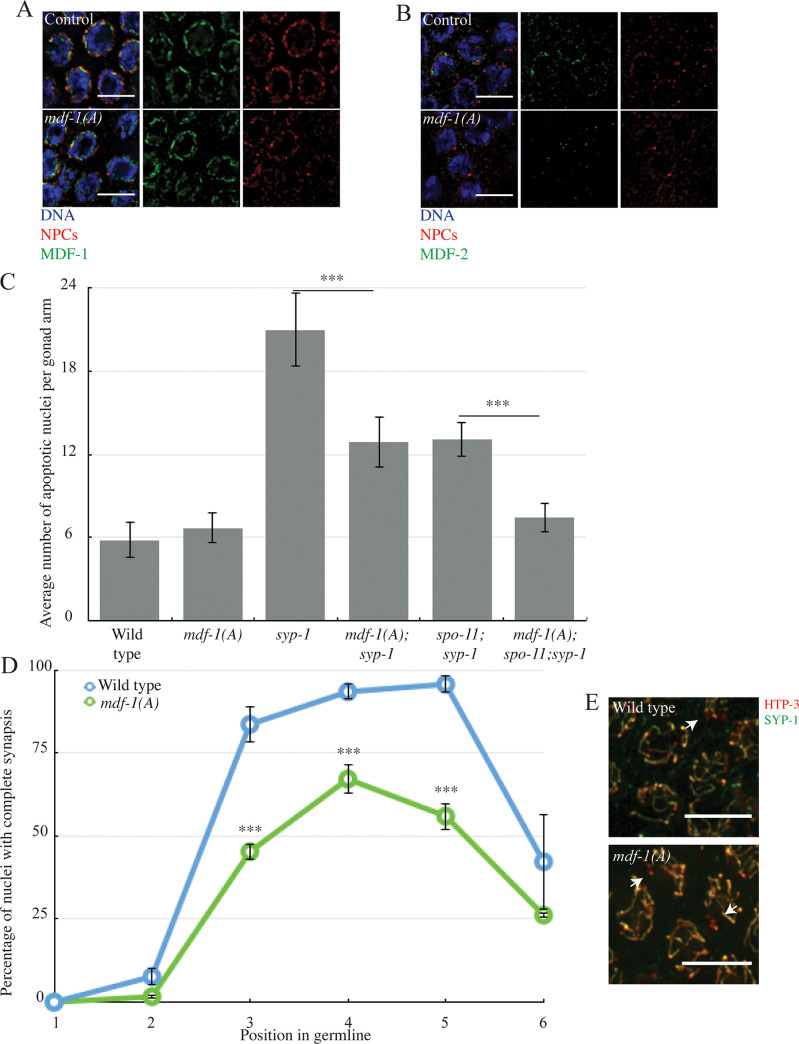
MDF-1^MAD-1^’s ability to interact with MDF-2^MAD-2^ is required to regulate and monitor synapsis. A. MDF-1^MAD-1^ (A) localizes at the nuclear periphery. B. MDF-2^MAD-2^ (green) does not co-localize with NPCs (red) at the nuclear periphery in *mdf-1*^*mad-1*^*(A)* mutants. Images are partial projections of meiotic nuclei stained to visualize DNA (blue). Bar: 5 μm. C. *mdf-1*^*mad-1*^*(A)* reduces germline apoptosis in *syp-1* and *spo-11;syp-1* mutants. A *** indicates a p value < 0.0001. D. Synapsis is reduced and delayed in *mdf-1*^*mad-1*^*(A)* mutants. E. Images of nuclei during synapsis initiation in wild-type and *mdf-1*^*mad-1*^*(A)* mutants stained to visualize SYP-1 and HTP-3. Arrows indicates unsynapsed chromosomes. Bar: 5 μm.

The correct localization of MDF-2^MAD-2^ in Δ*N-mdf-1*^*mad-1*^ deletion mutants led us to consider the effects on regulating and monitoring synapsis if MDF-1^MAD-1^ cannot bind MDF-2^MAD-2^. We used a point mutation in *mdf-1*^*mad-1*^, *mdf-1*^*mad-1*^*(P504A)*, which abolishes its ability to bind MDF-2^MAD-2^ (S1 Fig) [17]. We will refer to this allele as *mdf-1*^*mad-1*^*(A)* in this paper. First, we verified MDF-1^MAD-1^’s localization in meiotic germline nuclei in this background. After staining for MDF-1^MAD-1^ and NPCs (Fig 4A) or MDF-1^MAD-1^ and SUN-1 (S2C Fig), we were able to see that this point mutation does not affect the protein’s targeting to the nuclear periphery, similar to wild-type (Figs 4A and S2C) [9]. Next, we looked at MDF-2^MAD-2^ localization in this mutant background and were not able to detect the protein at the nuclear periphery (Fig 4B), similar to *mdf-1*^*mad-1*^*(av19)* mutants and *mdf-1*^*mad-1*^*(gk2)* null mutants (Fig 2). Thus, MDF-1^MAD-1^’s ability to bind MDF-2^MAD-2^ does not prevent MDF-1^MAD-1^’s localization to the nuclear periphery in meiotic germline nuclei but does affect MDF-2^MAD-2^’s.

We then investigated the implication of MDF-1^MAD-1^’s ability to bind MDF-2^MAD-2^ for the synapsis checkpoint (Fig 4C). We combined the *mdf-1*^*mad-1*^*(A)* mutation with the *syp-1* background. We were able to observe an intermediate reduction in the number of apoptotic nuclei, indicating that one of the two checkpoints is affected by the *mdf-1*^*mad-1*^*(A)* mutation (Fig 4C). To determine which checkpoint is affected, we generated the triple mutant *mdf-1*^*mad-1*^*(A); spo-11;syp-1*, which cannot activate the DNA damage checkpoint and only activates the synapsis checkpoint. Apoptosis was similar to wild-type in these triple mutants, indicating that MDF-1^MAD-1^’s ability to bind MDF-2^MAD-2^ is required for the synapsis checkpoint (Fig 4C).

Next, we investigated what effect this mutation had on synapsis. We observed that *mdf-1*^*mad-1*^*(A)* mutants exhibit a defect in SC assembly (Fig 4D, zones 2 and 3) and a reduction in the percentage of nuclei that complete synapsis (Fig 4D, zones 4 and 5, arrows in Fig 4E). To determine the downstream consequences of this defect, specifically whether it was accompanied by defects in crossover recombination, we analyzed nuclei in diakinesis, after the SC is disassembled. Wild-type diakinesis nuclei exhibit six DAPI staining bodies (Fig 6A and 6B), which are the six chromosome pairs linked by crossover recombination. *mdf-1*^*mad-1*^*(A)* mutants had a small but significant number of nuclei with greater than six DAPI staining bodies (5.7%) (Fig 6A and 6B), indicating that *mdf-1*^*mad-1*^(A) mutants also had defects in crossover recombination. Thus, MDF-1^MAD-1^’s ability to bind MDF-2^MAD-2^ is required to promote complete synapsis and crossover recombination.

Since this role in promoting synapsis and recombination was unexpected, we were concerned that the synapsis defects we observed might be the indirect consequence of aneuploidy from defects in mitosis earlier in the germline. To test this, we attempted to detect aneuploidy in *mdf-1*^*mad-1*^*(A)* mutant. We performed immunofluorescence with antibodies against HIM-8 to identify aneuploid nuclei that either had no HIM-8 staining or more than two HIM-8 foci (S5 Fig). We did not observe any nuclei with no HIM-8 or more than two HIM-8 signals in this mutant background, arguing against defects in ploidy and supporting a role for MDF-1^MAD-1^’s ability to bind MDF-2^MAD-2^ in regulating timely synapsis.

To further address this possibility, we scored apoptosis in *mdf-1*^*mad-1*^*(A)* single mutants. Defects in mitotic checkpoint function in mitotic germline nuclei can produce aneuploidy in meiotic nuclei that activate the DNA damage checkpoint and elevate apoptosis [18]. However, the level of apoptosis in *mdf-1*^*mad-1*^*(A)* single mutants was comparable to wild-type animals (Fig 4C), supporting our hypothesis that the synapsis defects we observe in *mdf-1*^*mad-1*^*(A)* mutant are not a consequence of defects in the mitotic region of the germline and are likely not severe enough to activate the DNA damage checkpoint, similar to other mutant backgrounds that exhibit asynapsis in a subset of meiotic nuclei [2,3]. In support of this interpretation, we localized PCH-2 in *mdf-1*^*mad-1*^*(A)* mutants and observed that it localized to meiotic chromosomes in the transition zone and was removed in late pachytene (S3 Fig), similar to control germlines and unlike other mutants that present more severe defects in recombination [13]. All together, these data indicate that MDF-1^MAD-1^’s ability to interact with MDF-2^MAD-2^ is important for MDF-2^MAD-2^ localization to the nuclear periphery but not for MDF-1^MAD-1^ targeting to the nuclear periphery. Further, this interaction is required to promote the synapsis checkpoint, synapsis and recombination. This is in contrast to *mdf-1*^*mad-1*^ null and *mdf-1*^*mad-1*^*(av19)* mutants, which promote the synapsis checkpoint but inhibit synapsis [9].

### MDF-2^MAD-2^’s ability to adopt the closed conformation is required to regulate and monitor synapsis

MDF-2^MAD-2^ is essential for the spindle checkpoint and the synapsis checkpoint. Its role in the spindle checkpoint has been extensively characterized [8]. MDF-2^MAD-2^ adopts two conformations, an open and a closed conformation, depending on whether it is binding other protein partners [19]. The open version is unbound and inactive in the spindle checkpoint. MDF-2^MAD-2^ adopts the closed version upon binding MDF-1^MAD-1^ [20,21] either at the nuclear envelope [22] or at unattached kinetochores [23–26]. Recent work has shown that when MDF-2^MAD-2^ is mutated so that it cannot convert to the closed conformation and remains locked in its open conformation, this mutant version of the protein cannot support the spindle checkpoint and is no longer detected at unattached kinetochores [27–29]. To evaluate the importance of this conversion for its meiotic role, we used a *mdf-2*^*mad-2*^ mutant that is locked in the open conformation *(mdf-2*^*mad-2*^*[V193N])* [28]; we will refer to this allele as *mdf-2*^*mad-2*^*-open*.

**Fig 5:**
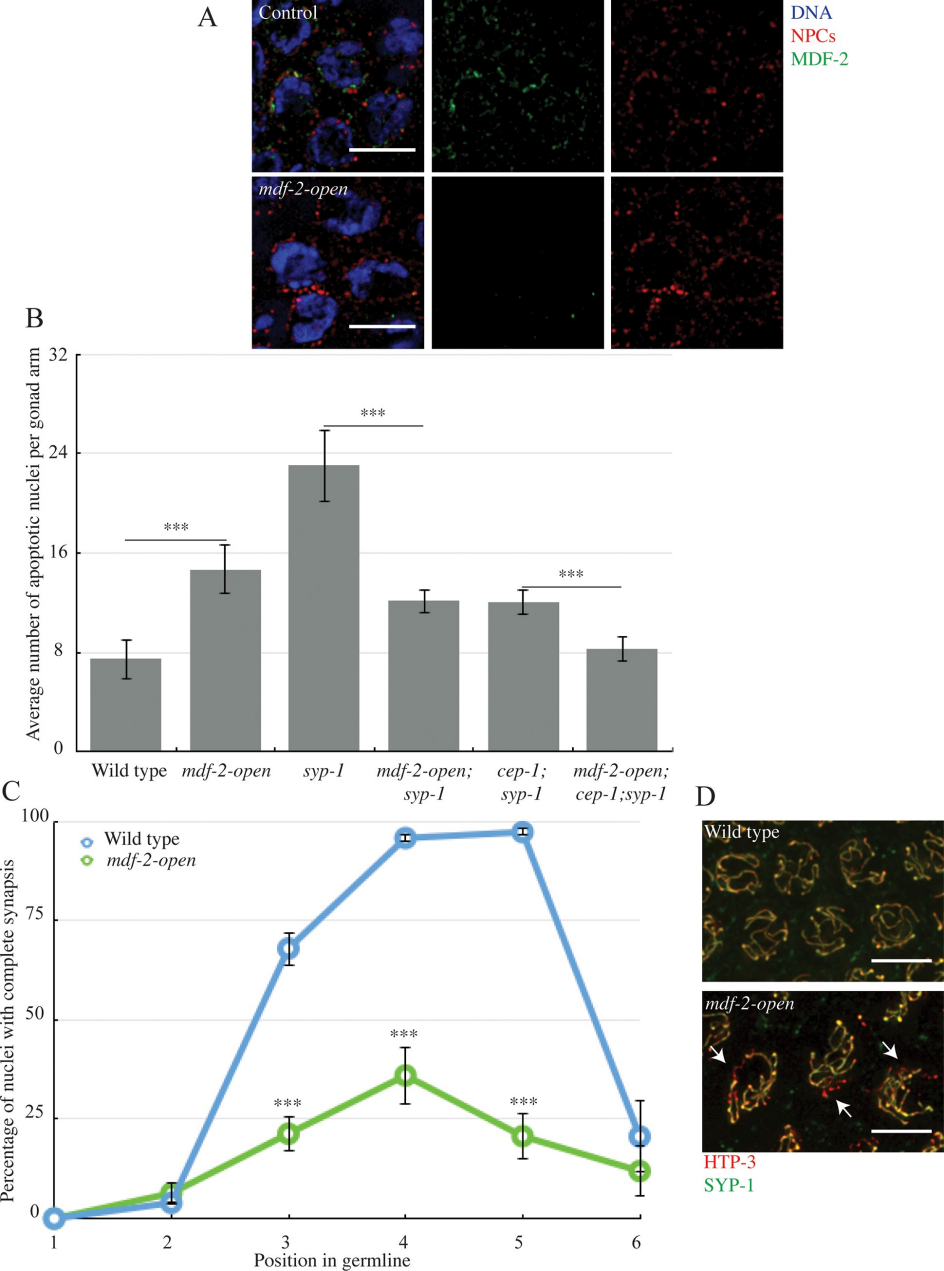
MDF-2^MAD-2^’s ability to adopt the closed conformation is required to regulate and monitor synapsis. A. MDF-2^MAD-2^ (green) does not co-localize with NPCs (red) at the nuclear periphery when the protein is locked in the open conformation. Images are partial projections of meiotic nuclei stained to visualize DNA (blue). Bar: 5 μm. B. *mdf-2*^*mad-2*^*-open* reduces germline apoptosis in *syp-1* and *cep-1;syp-1* mutants. A *** indicates a p value < 0.0001. C. Synapsis is reduced and delayed when MDF-2^MAD-2^ is locked in open conformation. A *** indicates a p value < 0.0001. D. Images of nuclei during synapsis initiation in wild-type and *mdf-2*^*mad-2*^*-open* mutants stained to visualize SYP-1 and HTP-3. Arrows indicates unsynapsed chromosomes. Bar: 5 μm.

First, we determined how this mutation affected the protein’s localization. When we stained germlines with antibodies against SUN-1 and MDF-2^MAD-2^ in *mdf-2*^*mad-2*^*-open* mutants, we could not detect the protein in meiotic nuclei (Fig 5A). Previous experiments have demonstrated that this mutation does not affect MDF-2^MAD-2^ stability [28]. However, we were concerned that the absence of staining was a consequence of the antibody not recognizing the mutant form of the protein. To verify that MDF-2^MAD-2^-open did not localize properly, we visualized a GFP-tagged version. Similar to the wild-type GFP::MAD-2, which cannot support checkpoint function [30], GFP:: MDF-2^MAD-2^-open did not localize to the nuclear periphery of transition zone (TZ) nuclei, as visualized by anti-SUN-1 staining (S6 Fig). However, in late pachytene (LP) nuclei, wild-type GFP::MAD-2 could be observed at the nuclear periphery but GFP:: MDF-2^MAD-2^-open could not (S6 Fig). Thus, MDF-2^MAD-2^’s ability to adopt the closed conformer is required for its localization to the nuclear periphery.

Next, we evaluated its role in the synapsis checkpoint. We introduced this mutation into *syp-1* mutants and assayed apoptosis. When compared to the *syp-1* single mutant background, *mdf-2*^*mad-2*^*-open*;*syp-1* double mutants exhibit an intermediate level of apoptosis (Fig 5B), indicating that either the synapsis checkpoint or the DNA damage checkpoint is affected. For these experiments, we used *cep-1* to prevent DNA damage checkpoint-induced apoptosis in *mdf-2*^*mad-2*^*-open* mutants. *cep-1* is the *C*. *elegans* ortholog of p53 and is required for the DNA damage response [31,32] but not the synapsis checkpoint [2]. We generated *mdf-2*^*mad-2*^*-open;cep-1;syp-1* triple mutants to clarify which checkpoint is affected. We observed levels of apoptosis in *mdf-2*^*mad-2*^*-open;cep-1;syp-1* triple mutants similar to wild-type animals, indicating the ability to adopt the closed conformation is required for the synapsis checkpoint (Fig 5B).

Having established that this mutant disrupted the synapsis checkpoint, we assessed its effect on synapsis (Fig 5C and 5D). We observed a reduction in the percentage of nuclei that completed synapsis in *mdf-2*^*mad-2*^ *open* mutants. This phenotype is more severe than the one observed for *mdf-1*^*mad-1*^*(A)* mutant (Fig 5D). In *mdf-1*^*mad-1*^*(A)* mutants, 70% of meiotic nuclei complete synapsis in zone 4, while in *mdf-2*^*mad-2*^*-open* mutants, only 40% do (Figs 4D, 5C and 5D).

**Fig 6:**
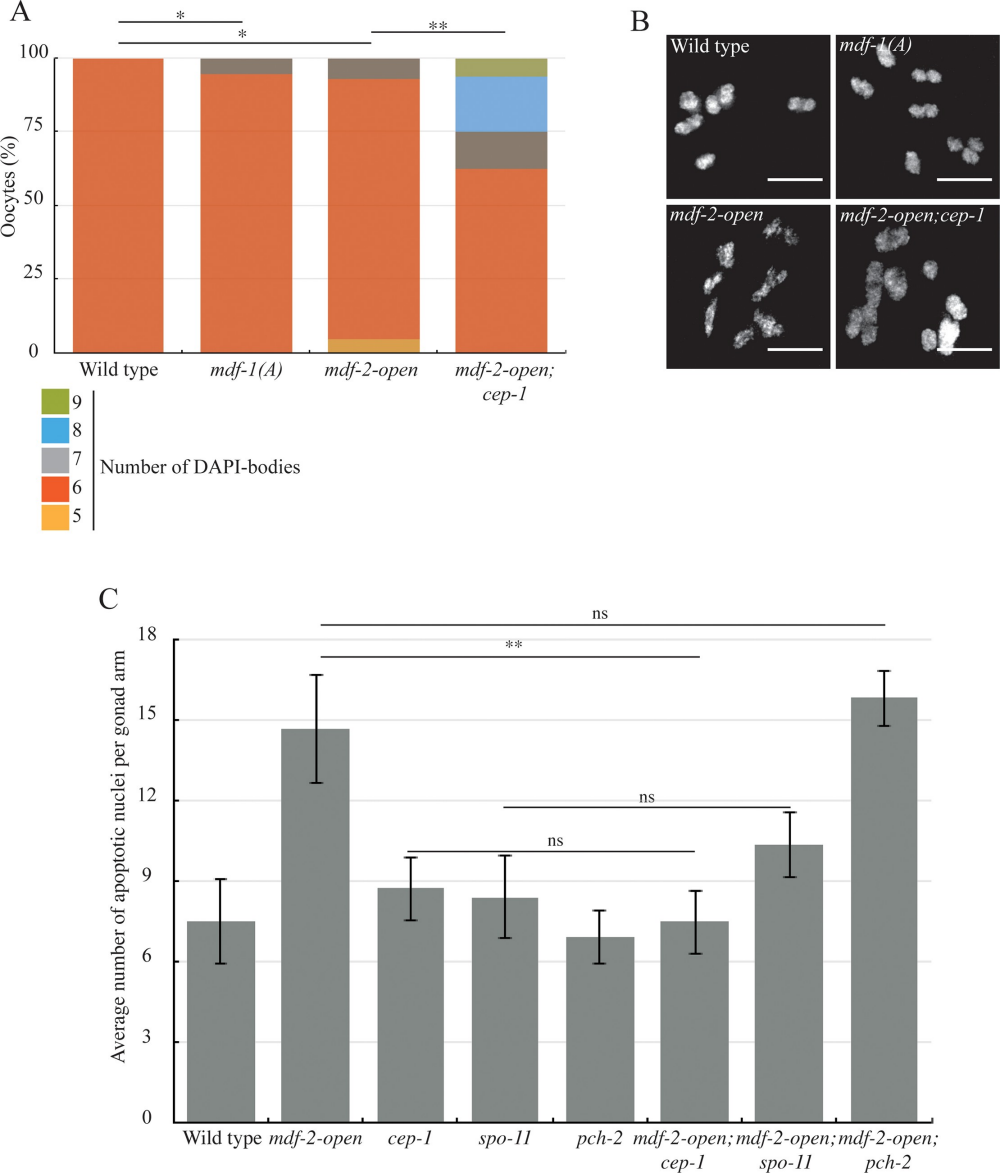
MDF-2^MAD-2^ locked in the open conformation activates the DNA damage checkpoint. A. *mdf-1(A)*, *mdf-2-open* and *mdf-2-open; cep-1* exhibit oocytes with more than 6 DAPI-staining bodies. A * indicates a p value < 0.05 and a ** indicates a p value < 0.01. B. Images of oocytes observed in wild-type and mutants. Bar 5 μm. C. Elevated apoptosis in *mdf-2*^*mad-2*^*-open* mutants relies on *cep-1* and *spo-11* but not *pch-2*. ns indicates not significant and a ** indicates a p value < 0.01.

Since complete synapsis is required for the proper progression of DNA repair and meiotic recombination, this defect in synapsis also results in an increase in DAPI staining bodies in diakinesis (Fig 6A and 6B). However, we were surprised to see that despite the more severe defect in synapsis, *mdf-2*^*mad-2*^*open* mutants had a similar defect in recombination as *mdf-1*^*mad-1*^(A) mutants (7.2% nuclei with achiasmate chromosomes). We reasoned that since *mdf-2*^*mad-2*^-*open* single mutants display elevated apoptosis (Fig 6B), the severe defect in synapsis may be activating the conserved DNA damage checkpoint. Indeed, when *mdf-2*^*mad-2*^*-open;cep-1* double mutants are generated and apoptosis assayed, the level of apoptosis is similar to *cep-1* single mutants and significantly lower than *mdf-2*^*mad-2*^*-open* single mutants (Fig 6C), indicating that *mdf-2*^*mad-2*^*-open* mutants activate the DNA damage checkpoint (S3 Fig). In contrast, when apoptosis was assayed in *mdf-2*^*mad-2*^*-open;pch-2* double mutants, we could detect no effect (Fig 6C). Further, when we monitor DAPI staining bodies in diakinesis in *mdf-2*^*mad-2*^*-open;cep-1* double mutants, significantly more nuclei exhibit more than six DAPI staining bodies, consistent with their removal by the DNA damage checkpoint in *mdf-2*^*mad-2*^*-open* single mutants (Fig 6A and 6B). To verify whether the elevated apoptosis in *mdf-2*^*mad-2*^*-open* mutants was specifically a response to unrepaired, programmed double strand breaks introduced by the meiotic enzyme SPO-11, we generated *mdf-2*^*mad-2*^*-open;spo-11 double* mutants and assayed apoptosis. *mdf-2*^*mad-2*^*-open;spo-11* had wild-type levels of apoptosis (Fig 6C), consistent with the DNA damage checkpoint being activated by unrepaired, programmed, meiotic double strand breaks.

We also monitored PCH-2 localization in *mdf-2*^*mad-2*^*-open* mutants. Consistent with the more severe defects in synapsis and recombination we detect in *mdf-2*^*mad-2*^*-open* mutants, PCH-2 localizes to meiotic chromosomes in the transition zone but its presence persists into late pachytene (S3 Fig), as we have reported for other mutants defective in synapsis and/or recombination [13].

Similar to our analysis of *mdf-1*^*mad-1*^*(A)* mutants, we wondered if some of the asynapsis in *mdf-2*^*mad-2*^*-open* mutants was the consequence of aneuploidy in meiotic nuclei. To assess this, we stained *mdf-2*^*mad-2*^*-open* meiotic nuclei with antibodies against the X chromosome PC protein, HIM-8. We detected nuclei that either contained no HIM-8 foci or more than two, indicating aneuploidy of the X chromosome (S5 Fig). When we quantified this defect, we observed it in 3% of meiotic nuclei. Therefore, some small proportion of unsynapsed chromosomes in meiotic nuclei are likely the product of aneuploidy and not strictly a defect in synapsis in *mdf-2*^*mad-2*^*-open* mutants. However, even if we assigned comparable rates of aneuploidy to the remaining five autosomes, this degree of aneuploidy is unlikely to explain the dramatic defect in synapsis that we observe in *mdf-2*^*mad-2*^*-open* mutants.

## Discussion

The spindle checkpoint, and the functional requirements of its essential factors, has been studied extensively [8]. We took advantage of these studies to test what aspects of MDF-1^MAD-1^ and MDF-2^MAD-2^ function are required for the regulation and monitoring of synapsis. Somewhat surprisingly, we found that a mutation that abolished MDF-1^MAD-1^’s association with the nuclear envelope [10] did not affect MDF-2^MAD-2^ localization (Figs 1A and 2), indicating that MDF-2^MAD-2^ may bind additional factors at the nuclear envelope besides MDF-1^MAD-1^ during meiosis. MDF-2^MAD-2^ has been shown to bind the insulin receptor and regulate its internalization dynamics in mice [33], raising the possibility that MDF-2^MAD-2^ may bind other factors at the nuclear envelope in other developmental contexts as well. Further, despite the more diffuse staining of MDF-1^MAD-1^ in meiotic nuclei when its N-terminus is deleted, the presence of MDF-2^MAD-2^ at the nuclear envelope still promotes the timely progression of synapsis (Fig 1D), suggesting that MDF-1^MAD-1^’s primary role in regulating synapsis is through control of MDF-2^MAD-2^.

This interpretation is borne out by our analysis of a *mdf-1*^*mad-1*^ mutant that no longer binds MDF-2^MAD-2^, *mdf-1*^*mad-1*^*(A*) [17]. This mutant protein is localized to the nuclear envelope (Fig 4A) but MDF-2^MAD-2^ is not (Fig 4B), indicating that although MDF-1^MAD-1^ may not be required for MDF-2^MAD-2^’s localization to the nuclear envelope, this interaction is required for MDF-2^MAD-2^’s presence inside the nucleus. This suggests a potential regulatory role for MDF-1^MAD-1^ in shuttling MDF-2^MAD-2^ into meiotic nuclei to carry out its role in regulating and monitoring synapsis. Indeed, we can detect MDF-2^MAD-2^ outside of meiotic nuclei visualized by SUN-1 staining in *mdf-1*^*mad-1*^*(A*) mutants (Figs 4B and S7).

We were surprised to observe that *mdf-1*^*mad-1*^*(A)* mutants, unlike *mdf-1*^*mad-1*^ null or *mdf-1*^*mad-1*^*(av19)* mutants, delay synapsis (Fig 4D). We ruled out the possibility that this was a consequence of the spindle checkpoint defect resulting in aneuploidy in meiotic cells (S5 Fig). Further, since *mdf-1*^*mad-1*^*(AAA)* mutants also have a spindle checkpoint defect [17] and do not affect synapsis (Fig 4D), we are comfortable attributing these phenotypes to a meiotic defect. These data suggest when MDF-2^MAD-2^ cannot bind MDF-1^MAD-1^, MDF-2^MAD-2^ may now be competent to bind additional meiotic factors, such as CMT-1 and/or PCH-2 [13, 14] that it is normally prevented from interacting with during meiosis, disrupting synapsis. Indeed, the degree of non-homologous synapsis we observe in *mdf-1*^*mad-1*^*(A)* mutants, ~4%, is similar to what is observed in *cmt-1* null mutants [14], consistent with this possibility. Given that MDF-2^MAD-2^ interacts with these factors during mitotic spindle checkpoint function [34], MDF-2^MAD-2^’s sequestration during meiosis may be an important regulatory event to promote meiotic synapsis.

Finally, we’ve shown that MDF-2^MAD-2^’s ability to adopt its closed conformation is important for its localization to the nuclear envelope (Fig 5A), its role in the synapsis checkpoint (Fig 5B) and its regulation of synapsis (Fig 5C). One of the proteins it complexes with to adopt its closed conformation is definitely MDF-1^MAD-1^, as demonstrated by MDF-2^MAD-2^ absence from the nuclear envelope in *mdf-1*^*mad-1*^*(A)* mutants (Fig 4B). However, MDF-2^MAD-2^’s continued presence at the nuclear envelope in Δ*N-mdf-1*^*mad-1*^ mutants (Fig 2) illustrates that MDF-2^MAD-2^ potentially complexes with some other factor at the nuclear envelope during meiotic prophase and this has important implications for the regulation and monitoring of synapsis in *C*. *elegans*. Identifying this factor is an important next step in understanding MDF-2^MAD-2^’s meiotic function.

Our previous model hypothesized that spindle checkpoint mutants regulate and monitor meiotic synapsis by assessing whether PCs at the nuclear envelope are synapsed [9], similar to their role in monitoring kinetochore attachment. However, it’s unlikely that the role of spindle checkpoint proteins in regulating and monitoring meiotic synapsis at unsynapsed PCs can be compared with their role at unattached kinetochores. First, while a mutation that prevents MDF-1^MAD-1^’s localization to the nuclear envelope, *ΔN-mdf-1*^*mad-1*^, abrogates the synapsis checkpoint (Fig 1C), it does not affect synapsis (Fig 1D), indicating that MDF-1^MAD-1^’s absence from the nuclear envelope does not affect the progression of synapsis. Further, the uncoupling of the regulation and monitoring of synapsis in Δ*N-mdf-1*^*mad-1*^ mutants indicates that its role in the checkpoint does not depend on its enrichment at the nuclear envelope, in direct contrast to our model. It is formally possible that MDF-1^MAD-1^’s dispensability in regulating synapsis is because of MDF-2^MAD-2^’s continued presence at the nuclear envelope in this mutant background (Fig 2). However, we do not favor this possibility based on MDF-2^MAD-2^’s absence at the nuclear envelope and the dramatic defect in synapsis we observe in *mdf-2*^*mad-2*^*-open* mutants (Fig 5A and 5C). If our model was correct, we might have predicted that *mdf-2*^*mad-2*^*-open* mutants would accelerate synapsis, similar to *mdf-1*^*mad-1*^*(av19)* and *mdf-1*^*mad-1*^ null mutants [9], which also fail to localize MDF-2^MAD-2^ at the nuclear envelope (Fig 2). Instead, these data suggest a more complicated role for spindle checkpoint proteins in regulating and monitoring synapsis than we had previously proposed. For example, multiple proteins at the nuclear periphery or the nuclear envelope, such as lamin [35] and SUN-1 [5] are required for accurate and timely synapsis in *C*. *elegans*. One possibility is that spindle checkpoint proteins, particularly MDF-2^MAD-2^, collaborate with lamin and/or through their documented interaction with SUN-1 [9] to contribute to the transmission of force through the nuclear envelope and regulate and monitor synapsis. Understanding this role may further expand the repertoire of spindle checkpoint proteins beyond their well-characterized roles in regulating the cell cycle and monitoring kinetochore attachment.

## Materials and methods

### Genetics and worm strains

The wild-type *C*. *elegans* strain background was Bristol N2 [36]. All experiments were performed on adult hermaphrodites at 20°C under standard conditions unless otherwise stated. Mutations and rearrangements used were as follows:

LG I: *cep-1(gk138)*

LG II: *bub-3(ok3437)*, *mln1 [mls14 dpy-10(e128)]*, *ltSi608[pOD1583/pMM30; pmdf-1*::*GFP*::*mdf1*::*mdf-1 3′UTR; cb-unc-119(+)]*, *ltSi609[pOD1584/pMM9; Pmdf-1*::*mdf-1(P504A)*::*mdf-1 3*’*UTR; cb-unc-119(+)]*, *ltSi620[pOD1595/pMM13; pmdf-1*::*GFP*::*mdf1(E419A*, *R420A*, *D423A)*::*mdf1 3*’*UTR; cb-unc-119(+)]*, *ltSi677 [pPLG034; Pmdf-1*::*GFP*::*mdf-1(Δ151–320)*::*mdf-1 3′UTR; cb-unc-119(+)]*, *ltSi1514[pPLG333; Pmdf-2*::*mdf-2 delta hairpin intron 4 V193N*::*mdf-2 3’UTR; cb-unc-119(+)]*, *ltSi1227[pPLG286; Pmdf-2*::*mdf-2 delta intron 4 V193N*::*GFP*::*mdf-2 3’UTR; cb-unc-119(+)]*

LG III: *unc-119(ed3)*

LG IV: *mdf-2(tm2190)*, *spo-11(ok79)*, *nT1[unc-*?*(n754let-*?*(m435)]*

LG V: *mdf-1(av19)*, *mdf-1(gk2)*, *syp-1(me17)*, *bcIs39[Plim-7*::*ced-1*::*gfp; lin-15(+)]*, *ieSi21 [Psun-1*::*sun-1*::*mRuby*::*sun-1 3’UTR + Cbr-unc-119(+)]*, *dpy-11(e224)*, *nT1[unc-*?*(n754let-*?*(m435)]*

### Quantification of germline apoptosis

Scoring of germline apoptosis was performed as previously described in [2]. L4 hermaphrodites were allowed to age for 22 h at 20°C. Live worms were mounted under coverslips on 1.5% agarose pads containing 0.2 mM levamisole for wild-type and moving strains or 0.1 mM levamisole for *dpy* strains. Minimum of 20 germlines were analyzed for each genotype by performing live fluorescence microscopy and counting the number of cells fully surrounded by CED-1::GFP. All experiments were performed three times. Significance was assessed using a paired *t*-test.

### Antibodies, immunostaining and microscopy

Immunostaining was performed on worms 20 to 24 f after L4 stage. Gonad dissection were performed in 1x EBT (250 mM Hepes-Cl, pH 7.4, 1.18 M NaCl, 480 mM KCl, 20 mM EDTA, 5 mM EGTA) + 0.1% Tween 20 and 20 mM sodium azide. An equal volume of 2% formaldehyde in EBT (final concentration was 1% formaldehyde) was added and allowed to incubate under coverslip for 5 min. The sample was mounted on HistoBond slides (75 x 25 x 1 mm from VWR), freeze-cracked, and immediately incubated in methanol at -20°C for 1 min and transferred to PBST (PBS with Tween20). After a total of 3 washes of PBST, the samples were incubated for 30 min in 1% bovine serum albumin diluted in PBST. A hand-cut paraffin square was used to cover the tissue with 50 μL of antibody solution. Incubation was conducted in a humid chamber at 4°C overnight. Slides were rinsed 3 times in PBST and incubated for 2 h at room temperature with fluorophore-conjugated secondary antibody solution at a dilution of 1:500. Samples were rinsed in PBST, DAPI stained in PBST (5 μg/mL) and rinsed a last time in PBST. Samples were then mounted in 12 μL of mounting media (20 M N-propyl gallate [Sigma- Aldrich] and 0.14 M Tris in glycerol) with a no. 1.5 (22 mm^2^) coverslip, and sealed with nail polish.

Primary antibodies were as follows (dilutions are indicated in parentheses). Rabbit anti-SYP-1 (1:500; [11]), chicken anti-HTP-3 (1:250; [3]), rabbit anti- MDF-2^MAD-2^ and anti- MDF-1^MAD-1^ (1:10000; [30]), Guinea pig anti-SUN-1 (1:500; [5]), rat anti-HIM-8 (1:2500; [37]) and goat anti-GFP (1:10000; [38]). Antibodies against SYP-1 were provided by A. Villeneuve (Stanford University, Palo Alto, CA). Antibodies against HTP-3 and HIM-8 were provided by A. Dernburg (University of California Berkley/E.O. Lawrence Berkley National Lab, Berkley, CA). Antibodies against MDF-1^MAD-1^ and MDF-2^MAD-2^ were provided by A. Desai (Ludwig Institute/University of California, San Diego, CA). Antibodies against GFP were provided by S. Strome (University of California, Santa Cruz, CA). Antibodies against SUN-1 were provided by Verena Jantsch (Max Perutz Laboratories, University of Vienna).

Secondary antibodies were Cy3, Cy5 and Alexa Fluor 488 anti-mouse, anti-rabbit, anti-guinea pig, anti-rat and anti-chicken (1:250; Jackson ImmunoResearch Laboratories, Inc.)

Quantification of synapsis was performed with a minimum of three whole germlines per genotype as in [37] on animals 24 h after L4 stage. The gonads were divided into six equal-sized regions, beginning at the distal tip of the gonad and progressing through the end or late pachytene.

All images were acquired at room temperature using a Delta-Vision Personnal DV system (GE Healthcare) equipped with a 100x NA 1.4 oil immersion objective (Olympus), resulting in an effective xy pixel spacing of 0.064 or 0.040 μm. Images were captured using a charge-coupled device camera (Cool-SNAP HQ; Photometrics). Three-dimensional images stacks were performed using functions in the softWoRx software package. Projections were calculated by a maximum intensity algorithm. Composite images were assembled, and some false coloring was performed with Fiji and Photoshop software (Adobe).

## Supporting information

S1 Fig Summary of *mdf-1*^*mad-1*^ mutants studied in this paper. A. Cartoon of the different *mdf-1*^*mad-1*^ mutants studied in this paper. B. Summary of observed phenotypes.
(EPS)

S2 Fig MDF-1^MAD-1^(A), but not ΔN-MDF-1^MAD-1^, localizes to the nuclear periphery. A. Line intensity analysis was performed across a single Z section from the center of an individual nuclei in control and *ΔN-mdf-1*^*mad-1*^ mutants stained to visualize NPCs (red) and MDF-1^MAD-1^ (green), as defined by the yellow line. Bar: 2 μm. B. A plot of the intensities of the two wavelengths (red = wavelength 605, green = wavelength 525) on a pixel by pixel basis of the same images in A. The Pearson coefficient of correlation for the control nucleus is 0.7596 and the Pearson coefficient of correlation for the *ΔN-mdf-1*^*mad-1*^ mutant nucleus is 0.2613. C. ΔN-MDF-1^MAD-1^ (green) localizes diffusely in the cytoplasm of meiotic nuclei. MDF-1^MAD-1^ (A) (green) localizes at the nuclear periphery. Images are partial projections of meiotic nuclei stained to visualize DNA (blue) and SUN-1 (red). Bar: 5 μm.
(EPS)

S3 Fig PCH-2 localization is extended in *mdf-2^mad-2^-open* mutants. A. PCH-2 (green) localization is similar to wild-type in *mdf-1*^*mad-1*^*(A)* and *ΔN-mdf-1*^*mad-1*^ mutants but is extended into late pachytene in *mdf-2*^*mad-2*^*-open* mutants. Images are full projections of top rows of meiotic nuclei stained to visualize DNA (magenta) Bar: 50 μm. B. Example of nuclei in transition zone and late pachytene. Bar: 5 μm.
(EPS)

S4 Fig BUB-1 is localized in the cytoplasm of *ΔN-mdf-1^mad-1^*, *mdf-1^mad-1^(A)* and *mdf-2^mad-2^-open* mutants. Images are partial projections of meiotic nuclei stained to visualize DNA (blue) and BUB-1 (green). Bar: 5 μm.
(EPS)

S5 Fig Aneuploidy is observed in *mdf-2^mad-2^-open* but not *mdf-1^mad-1^(A)* mutants. A. Meiotic nuclei exhibit aneuploidy in *mdf-2*^*mad-2*^*-open* mutants but not in *mdf-1*^*mad-1*^*(A)* mutants. B. Example of nuclei exhibiting aneuploidy in *mdf-2*^*mad-2*^*-open* mutants. Images are projections of meiotic nuclei stained to visualize DNA (blue) and HIM-8 protein (red). Arrows indicates nuclei with no HIM-8 foci. Bar: 5 μm.
(EPS)

S6 Fig GFP::MDF-2^MAD-2^ is detected at the nuclear periphery in pachytene meiotic nuclei while GFP::MDF-2^MAD-2^ open is not. Images are partial projections of meiotic nuclei stained to visualize DNA (blue), SUN-1 (red) and MDF-2^MAD-2^ (green). Bar: 5 μm.
(EPS)

S7 Fig MDF-2^MAD-2^ is present in the cytoplasm in *mdf-1^mad-1^(A)* mutants. Images are partial projections of meiotic nuclei stained to visualize DNA (blue), SUN-1 (red) and MDF-2^MAD-2^ (green). Bar: 5 μm.
(EPS)
