# Supplementary material for: Mad1’s ability to interact with Mad2 is essential to regulate and monitor meiotic synapsis in C. elegans
Source: PLoS Genet. 2021 Nov 11;17(11):e1009598. doi: 10.1371/journal.pgen.1009598 (PMC8610272; doi:10.1371/journal.pgen.1009598)
Supplement: Attachment — Submitted filename: coverletterDevigneandBhalla.pdf [file pgen.1009598.s008.pdf]

Needhi Bhalla, Ph.D.
Professor
Department of Molecular, Cell and Developmental Biology
Sinsheimer Labs
University of California, Santa Cruz
Santa Cruz, CA 95064
phone: 831.459.1319
fax: 831.459.3139
email: nbhalla@ucsc.edu
September 27, 2021

Dear JoAnne-

Thank you for the careful review and consideration of our manuscript, “Mad1’s ability to interact with Mad2 is essential to regulate and monitor meiotic synapsis in *C. elegans.”* We are encouraged by the reviewers’ comments on our manuscript and appreciate their thoughtful and helpful suggestions to make this a stronger, more accessible and compelling story. In particular, we removed the analysis of chromosome mobility, strengthened our analysis of Mad-1’s and Mad-2’s localization in some mutant backgrounds and added additional analysis of the effect of some mutations on crossover recombination. Here, we present a point-by point response. **Our responses are in bold.**

Regards,

Needhi Bhalla

Reviewer #1:

Figure 1. This piece of data is rather superficial, and it does not seem to add major information to the work as is. The authors claim that MAD-1 and BUB-3 regulate PCM, but by looking at the numbers, it is clear that their contribution is not really major. They could operate redundantly, and therefore, unless there is not a valid reason not to build the strain (or by RNAi), this analysis should be repeated under contemporary depletion of mad-1 and bub-3. Moreover, the number of nuclei analysed for each genotype is really low: do "3 to 5 nuclei" provide a significant frame to the analysis, sufficient to extrapolate any solid conclusions? Hence, the authors should reinforce this analysis.

We performed analysis of 3-5 nuclei per germline and analyzed 2-3 germlines per genotype, resulting in a total analysis of 8-10 nuclei per genotype. However, this and other reviewers pointed out that this analysis did not seem to add much to the conclusions of the paper. For this reason, we have removed this data.

Figure 2. In the example provided in Fig. 2A I can still see MAD-1 at the nuclear periphery and in some areas even still co-localizing with the NPCs. One nucleus is not representative of a whole portion of the gonad, and therefore a larger inset showing should be provided. A staining with a marker surrounding the whole nuclear envelope should be employed (eg. SUN-1), as this would simplify the discrimination between the nuclear membrane and the interior. I mentioned SUN-1, but any marker with a similar localization would suffice.

We have provided additional larger fields and reproduced staining using antibodies against SUN-1 for this figure to provide further evidence that this mutation reduces localization to the nuclear periphery. Further, we have presented line intensity analysis and colocalization analysis to further support our interpretation (Figures S2A and B).

-Figure 5. As for Fig. 2A, also in 5A-B having a marker that stains the nuclear envelope would help (especially in 5B). The anti-MAD-2 still detects foci surrounding both the chromatin and the NPCs and therefore, to support their claims the authors should provide more convincing staining examples.

We have provided additional larger fields and reproduced staining using antibodies against SUN-1 for this figure.

I would not define as "dramatic" the difference in zone 2 (Fig. 5D, line 304) and I also do not think it is appropriate to use the term "delayed" here. In fact, a "delayed" synapsis would imply that at some point the mad-1(A) reached full synapsis, which instead it never happens. Therefore, it should simply be stated in here that mad-1(A) displays a defect in SC assembly.

We have changed the discussion of the synapsis defect in *mad-1(A)* mutants to: "We observed that *mad-1(A)* mutants exhibit a defect in SC assembly (Figure 4D, zones 2 and 3) and a reduction in the percentage of nuclei that complete synapsis (Figure 4D, zones 4 and 5, arrows in Figure 4E)."

Moreover, by looking at the examples provided in Fig. 5E, the unsynapsed regions seem to be very small and the authors should make sure that, if my impression is correct, this is clearly specified in the text. Considering the images, to me it looks like mad-1(A) is largely proficient in establishing the SC globally, and that only small portions of chromosomes remain unsynapsed. If instead this phenotype is more severe than it looks from the pictures, then it should be reflected in the presence of achiasmatic chromosomes in the diakinesis nuclei: have the authors looked at this? If not, they should.

We performed analysis of achiasmate chromosomes in diakinesis. Consistent with the defects in synapsis we observe, we also detect an increase in the number of DAPI staining bodies, consistent with achiasmate chromosomes. These data are now shown in Figure 6 and discussed in the text.

Furthermore, since they both have a checkpoint activation, I would like to see whether the expression/localization of PCH-2 is altered in mad-1(A) and DN-mad-2.

***mad-1(A)* and *ΔN-mad-1* mutants fail to activate the checkpoint and PCH-2 localizes to meiotic chromosomes in both *mad-1(A)* and *ΔN-mad-1* mutants, resembling wildtype localization. This data is now in Figure S3 and discussed in the text.**

Figure 6. Line 353: I suppose the authors used the mad-2(V193N) that has been already characterized elsewhere? If this is the case, please include the appropriate reference (Lara-Gonzalez et al.; 2021).

We included this reference earlier and have now included it after the introduction of this mutant.

Lines 356-359: this whole section is a bit over-emphasized, since this mutant was previously characterized, and it was already shown that this “locked-open” version of MAD-2 is not detectable in the nucleus (Lara-Gonzalez et al.; 2021). Therefore it´s not surprising that this holds true also in the germ cells.

We have rewritten this section to condense it but still make it accessible to readers not familiar with the spindle checkpoint field. We are not aware of the data that MAD-2-open is not in the nucleus. According to Lara-Gonzalez et. al 2021, MAD-2-open fails to localize to unattached kinetochores during mitosis (Figure 2A) but they did not report its localization when the nuclear envelope is present, as far as we could see. As such, it's absence in germ cells seems important to report, particularly in the context of its meiotic phenotype.

Lines 365-371: mdf-2 is sitting at the genetic position -6.82 on Ch. IV and spo-11 is at +4.89, meaning that they are almost 12 cM away from each other: this to me does not really qualify as “closely linked”. The authors should build the double with spo-11 and re-assess apoptosis to make sure that it is comparable to the cep-1 data. Most importantly, given the robust activation of the DNA damage checkpoint in the mad-2-open mutants, the removal of SPO-11 will univocally show, if that´s the case, that the persistent damage due to the extensive asynapsis is what triggers the activation of the checkpoint. The authors do contemplate this possibility in the text, however it should be directly proved.

We constructed the *mad-2-open;spo-11* double mutant and assayed apoptosis in this strain. These data are now shown in Figure 6 and discussed in the text.

It would be nice to see a PCH-2 staining in the mad-2-open mutants as well.

We've performed this experiment. These data are now shown in Figure S3 and discussed in the text.

The extent of synapsis defect in the nuclei of the mad-2-open seem to be much more pronounced compared to the mad-1(A): the authors should perform the analysis of DAPI-bodies in the diakinesis nuclei of the mad-2-open mutants.

We performed the analysis of DAPI bodies in *mad-2-open* mutants as well and find an increase in achiasmate chromosomes in this mutant background as well. These data are now shown in Figure 6 and discussed in the text.

The fact that in the double with cep-1 there is already a full abrogation of the increase in the apoptosis levels, suggests that the major trigger for the checkpoint activation involves DNA damage, however I would also like to see if removing pch-2 from the mad-2-open mutant would make any difference.

We generated this double mutant and scored apoptosis in this background. Loss of *pch-2* does not affect apoptosis in *mad-2-open* mutants. This data is now included in Figure 6 and discussed in the text.

As for any control mechanism, I would expect that if in a certain mutant background there is a substantial activation of a checkpoint, then suppressing it, should elicit formation of aberrant gametes. For this reason, DAPI-bodies analysis in the diakinesis nuclei should be conducted also in the mad-2-open; cep-1 doubles, as it would be interesting to assess which is the biological significance of preventing activation of these checkpoints during meiotic prophase I.

We performed the analysis of DAPI bodies in *mad-2-open;cep-1* double mutants as well and find that both the number of achiasmate chromosomes and the number of nuclei with achiasmate chromosomes increases, consistent with the DNA damage response playing an important role maintaining gamete quality in *mad-2-open* mutants. These data are now shown in Figure 6 and discussed in the text.

Last but not least: I am aware that mad-1 and mad-2 have been used to refer to mdf- 1/2 for simplicity, but I also believe that is important to stick with the nomenclature in order to avoid confusion. The fact that this has been done in a couple of different studies does not necessarily mean that is appropriate. Since mdf-1/2 is the assigned name to these genes, either the authors request a name change to Wormbase or they should keep their correct identification names.

We have used the assigned nomenclature for *mdf-1*^{mad1} and *mdf-2*^{mad2} in the manuscript, using superscripts to indicate the more common names for these genes in the field.

Reviewer #2:

The current Figures show just one representative nucleus of each mutant without quantification. I would suggest that the authors show zoomed-out views capturing multiple germline nuclei and also quantify the fluorescence intensity at the nuclear periphery using MAD-2::GFP, like the analysis performed in Figure S3H of Lara-Gonzalez et al., 2019.

We have provided additional larger fields and reproduced staining using antibodies against SUN-1, as suggested by Reviewer #1. Further, we have presented line intensity analysis and colocalization analysis to further support our interpretation (Figures S2A and B). We did not perform analysis with GFP::MAD-2 since this tagged transgene is not fully functional, does not localize to meiotic nuclei in the transition zone (Figure S6) and does not support checkpoint function, unlike GFP::MAD-1, as depicted in Figure S3H in Lara-Gonzalez et. al., 2019.

Some of the details in the Discussion regarding the localization and function of Mad2 need further clarification. It appears that MAD-2 is absent in mad-1(A) mutants, and the authors speculated that MAD-1 might be involved in shuttling MAD-2 into the meiotic nuclei (line 430). Is MAD-2 found in the cytoplasm within the germline? Can the authors clarify what they mean by “a gain of function” (lines 439-441) when MAD-2 is not present in the nucleus in mad-1(A) mutants? Is there evidence showing that MAD-2 is upregulated in mad-1(A) mutants as suggested in line 440?

We have provided data to show the MAD-2 staining in the cytoplasm in *mad-1(A)* mutants in Figures 4B and S7. We have attempted to make this section clearer. We have removed the reference to a gain of function and changed the sentence to: “These data suggest when MAD-2 cannot bind MAD-1, MAD-2 may now be competent to bind additional meiotic factors, such as CMT-1 and/or PCH-2 (Deshong et al., 2014; Giacopazzi et al., 2020) that it is normally prevented from interacting with during meiosis, disrupting synapsis.” We are not suggesting that MAD-2 is upregulated in mad-1(A) mutants but now more available to bind other meiotic proteins, such as PCH-2 and CMT-1.

Is MAD-2 protein present in mad-2 open mutants? Can the MAD-2 antibody recognize the open form? Can the authors explain the synapsis defects in these mutants as opposed to the accelerated synapsis in mad-2 null?

Lara-Gonzalez et. al. 2021 showed that this mutation does not affect the stability of MAD-2. The antibody against MAD-2 that we used, generated by the Desai/Oegema labs, is a polyclonal antibody generated against the whole protein. To test whether this antibody may not accurately recognize the MDF-2^{V193N}, we localized a GFP-tagged version of this mutant protein and found that it also did not localize to meiotic nuclei (Figure S6). Unfortunately, we never were able to assay synapsis in *mad-2* null mutants given the disorganization of the germline observed in this mutant background, a phenotype that is not observed in *mad-2-open* mutant.

Here are other points:
1. The first paragraph of the Results section is probably better suited for the Introduction. This background information can be streamlined with the existing Introduction.

This paragraph has been removed.

2. Regarding the results shown in Figure 1, it is unclear how the reduced frequency of PCM can explain the accelerated synapsis in mad-1 and bub-3 mutants. Isn't the PCM required for robust synapsis?

This and other reviewers pointed out that this analysis did not seem to add much to the conclusions of the paper. For this reason, we have removed this data. However, to address your question, PCMs contribute to synapsis but their particular role (assessing homology in preparation for synapsis, removing non-homologous interactions in preparation for synapsis, or some combination of the two) in the process in unclear.

3. In lines 191-193, the synaptonemal complex has already been defined in the Introduction (lines 66-68).

We have removed the definition of the synaptonemal complex in lines 191-193

4. In lines 205-207, please change the verbs to past tense.

We have made this change.

5. After line 242, adding a sentence summarizing the results will be helpful.

We have added the following line: "Altogether, these results indicate that while MAD-1 is required for MAD-2's localization in meiotic nuclei, MAD-1's localization to the nuclear envelope is not."

6. Is BUB-1 or BUB-3 localization known in the germline? This knowledge will help interpret the results using MAD-1(AAA) and the role of Bub3 in synapsis checkpoint.

We have localized both BUB-1 in the germline and it is unaffected in the mutants we analyzed. These data are now included in Figure S4 and discussed in the text. We did not analyze BUB-3 localization since it was not a focus of this paper.

7. For the experiments shown in Figure 6B using cep-1, please mention it was previously shown that the synapsis checkpoint is independent of CEP-1 (Bhalla and Dernburg, 2005), as it may not be apparent to readers who are outside of this immediate field.

We have included this change. The new sentence is: "*cep-1* is the *C. elegans* ortholog of p53 and is required for the DNA damage response (Derry et al., 2001; Schumacher et al., 2001) but not the synapsis checkpoint (Bhalla and Dernburg, 2005)."

8. For Figure S1, I would suggest switching the order between MAD-1(A) and MAD-1(AAA), following the order of experiments described in the main text.

We have made this change.

Reviewer #3:

1) The data in Figure 1 was not tied well together with the mutant analysis. It read as tying up loose ends from a previous manuscript without a strong rationale for how it fits with the other data in the manuscript.

This and other reviewers pointed out that this analysis did not seem to add much to the conclusions of the paper. For this reason, we have removed this data.

2) In Figure 2A, it does look like there is some co-localization in the deltaN-mad-1 mutant. Can the authors perform a quantitative analysis on the percent co-localization in wildtype and mutant?

We reproduced staining using antibodies against SUN-1 to provide further evidence that this mutation reduces localization to the nuclear periphery. We also performed intensity analysis and colocalization analysis of NPCs and MAD-1 in individual nuclei in control and *ΔN-mad-1* mutants. These data are now included in Figure S2A and B.

3) In Figure 2C, the delta-N-mad-1; syp-1 double mutant has more apoptosis than the spo-11; syp-1 double mutant. Is this difference significant? If so, doesn't the result suggest that the DNA damage checkpoint is also being triggered in the delta-N-mad-1; syp-1 double?

This result is significant. However, this likely either reflects the fact that this mutation does not affect meiotic events like synapsis or the variability in the number of apoptotic nuclei we observe in some mutant backgrounds. This second point is often common in our experiments and not necessarily biologically relevant, which is why we focus on the difference between *syp-1* mutants and *syp-1;ΔN-mad-1* double mutants in our analysis.

4) Why is Figure S3 a supplemental figure. It seems important and should be part of Figure 6.

We have moved this from Figure S3 and included it with Figure 6.

5) What epitope does the MAD-2 antibody recognize and can this account for why a mad-2-open mutant version does not show MAD-2 staining?

The antibody against MAD-2 that we used, generated by the Desai/Oegema labs, is a polyclonal antibody generated against the whole protein. To test whether this antibody may not accurately recognize the MDF-2^{V193N}, we localized a GFP-tagged version of this mutant protein and found that it also did not localize to meiotic nuclei. These data are now in Figure S6.

6) Do the mad-1 mutants have defects in PCMs (for example, the delta-N-mad-1 and the mad-1-A)? What about the mad-2-open mutant?

Because we removed this data based on the concerns of this and other reviewers, we did not include these data.

7) The story ended without a strong understanding of why the different mutants gave the different phenotypes and how the mutant analysis could be tied together in a model for how MAD-1 and MAD-2 function in synapsis and the synapsis checkpoint. Can the authors provide a more comprehensive model?

We have added the following sentence to the concluding paragraph: "Instead, these data suggest a more complicated role for spindle checkpoint proteins in regulating and monitoring synapsis than we had previously proposed. For example, multiple proteins at the nuclear periphery or the nuclear envelope, such as lamin (Link et al., 2018) and SUN-1 (Penkner et al., 2007a, 2007b) are required for accurate and timely synapsis in *C. elegans*. One possibility is that spindle checkpoint proteins, particularly MDF-2^{MAD-2}, collaborate with lamin and/or through their documented interaction with SUN-1 (Bohr et al., 2015) to contribute to the transmission of force through the nuclear envelope and regulate and monitor synapsis. Understanding this role may further expand the repertoire of spindle checkpoint proteins beyond their well-characterized roles in regulating the cell cycle and monitoring kinetochore attachment."
