# Supplementary material for: Mad1’s ability to interact with Mad2 is essential to regulate and monitor meiotic synapsis in C. elegans
Source: PLoS Genet. 2021 Nov 11;17(11):e1009598. doi: 10.1371/journal.pgen.1009598 (PMC8610272; doi:10.1371/journal.pgen.1009598)
Supplement: Peer review history

# Peer review: Decision Letter 0

**DOI:** 10.1371/journal.pgen.1009598.r001

**Authors:** Gregory P. Copenhaver, JoAnne Engebrecht

6 Jun 2021

Dear Needhi,

Thank you very much for submitting your Research Article entitled 'Mad1’s ability to interact with Mad2 is essential to regulate and monitor meiotic synapsis in C. elegans' to PLOS Genetics.

The manuscript was fully evaluated at the editorial level and by three independent peer reviewers. The reviewers appreciated the attention to an important problem, but raised some substantial concerns about the current manuscript. Based on the reviews, we will not be able to accept this version of the manuscript, but we would be willing to review a much-revised version. We cannot, of course, promise publication at that time.

Should you decide to revise the manuscript for further consideration here, your revisions should address the specific points made by each reviewer. We will also require a detailed list of your responses to the review comments and a description of the changes you have made in the manuscript. It will be particularly important to quantitate your cytological analyses and perform the additional analyses the reviewers requested to confirm and strengthen your conclusions.

If you decide to revise the manuscript for further consideration at PLOS Genetics, please aim to resubmit within the next 90 days, unless it will take extra time to address the concerns of the reviewers, in which case we would appreciate an expected resubmission date by email to plosgenetics@plos.org.

If present, accompanying reviewer attachments are included with this email; please notify the journal office if any appear to be missing. They will also be available for download from the link below. You can use this link to log into the system when you are ready to submit a revised version, having first consulted our Submission Checklist.

To enhance the reproducibility of your results, we recommend that you deposit your laboratory protocols in protocols.io, where a protocol can be assigned its own identifier (DOI) such that it can be cited independently in the future. Additionally, PLOS ONE offers an option to publish peer-reviewed clinical study protocols. Read more information on sharing protocols at https://plos.org/protocols?utm_medium=editorial-email&utm_source=authorletters&utm_campaign=protocols

Please be aware that our data availability policy requires that all numerical data underlying graphs or summary statistics are included with the submission, and you will need to provide this upon resubmission if not already present. In addition, we do not permit the inclusion of phrases such as "data not shown" or "unpublished results" in manuscripts. All points should be backed up by data provided with the submission.

While revising your submission, please upload your figure files to the Preflight Analysis and Conversion Engine (PACE) digital diagnostic tool.  PACE helps ensure that figures meet PLOS requirements. To use PACE, you must first register as a user. Then, login and navigate to the UPLOAD tab, where you will find detailed instructions on how to use the tool. If you encounter any issues or have any questions when using PACE, please email us at figures@plos.org.

PLOS has incorporated Similarity Check, powered by iThenticate, into its journal-wide submission system in order to screen submitted content for originality before publication. Each PLOS journal undertakes screening on a proportion of submitted articles. You will be contacted if needed following the screening process.

To resubmit, use the link below and 'Revise Submission' in the 'Submissions Needing Revision' folder.

[LINK]

We are sorry that we cannot be more positive about your manuscript at this stage. Please do not hesitate to contact us if you have any concerns or questions.

Yours sincerely,

JoAnne Engebrecht

Guest Editor

PLOS Genetics

Gregory P. Copenhaver

Editor-in-Chief

PLOS Genetics

Reviewer's Responses to Questions

**Comments to the Authors:**

**Please note here if the review is uploaded as an attachment.**

Reviewer #1: In the manuscript by Devigne and Bhalla, the authors describe novel roles for

Caenorhabditis elegans mdf-1/Mad1 and mdf-2/Mad2 (called throughout the

manuscript mad-1 and mad-2 respectively) in promoting the timely establishment and

maintenance of the synaptonemal complex and in monitoring the synapsis checkpoint.

The authors report on the characterization of different mutant alleles of these factors

and their impact on multiple aspects of mad-1/2-driven roles in the germ line.

They show that while recruitment of MAD-1 at the nuclear periphery is required for the

synapsis checkpoint, it is dispensable for MAD-2 localization at this site and does not

perturb synapsis. However, interaction between MAD-1/2 seems to be important to preserve

functionality of the synapsis checkpoint and to ensure normal kinetics of the SC

polymerization.

Finally, a mutation that prevents MAD-2 from acquiring its “closed”, functionally active,

conformation necessary to exert its roles within the spindle checkpoint, also bears

functional implications during gametogenesis, causing a profound perturbation in the

establishment of meiotic chromosome synapsis.

The manuscript is clearly written and the experimental design is appropriately crafted,

although it lacks depth in some parts. It is also worth mentioning that all the mutant

alleles shown in this work were already generated and characterized in detail

elsewhere (although in mitotic cells), where the consequences of such mutations were

biochemically and genetically dissected (Lara-Gonzalez et al.; 2021). Therefore, the

novelty of this works relies on the effect that these mutations exert in the germ line.

That being said, while I believe that this analysis illuminates novel and interesting

aspects of the roles exerted by MAD-1/2 in the germline, yet some of the findings

require further experimental corroboration. Some of the authors ‘claims need to be

strengthened by the inclusion of further experiments and the adequate controls, which

I think are lacking in some sections of this work, to comply with the quality standards

required by PLoS Genetics.

-Figure 1. This piece of data is rather superficial, and it does not seem to add major

information to the work as is. The authors claim that MAD-1 and BUB-3 regulate

PCM, but by looking at the numbers, it is clear that their contribution is not really major.

They could operate redundantly, and therefore, unless there is not a valid reason not

to build the strain (or by RNAi), this analysis should be repeated under

contemporary depletion of mad-1 and bub-3. Moreover, the number of nuclei analysed

for each genotype is really low: do “3 to 5 nuclei” provide a significant frame to the

analysis, sufficient to extrapolate any solid conclusions? Hence, the authors should

reinforce this analysis.

-Figure 2. In the example provided in Fig. 2A I can still see MAD-1 at the nuclear

periphery and in some areas even still co-localizing with the NPCs. One nucleus is not

representative of a whole portion of the gonad, and therefore a larger inset showing should be provided.

A staining with a marker surrounding the whole nuclear envelope should be employed (eg. SUN-1), as this would simplify the

discrimination between the nuclear membrane and the interior. I mentioned SUN-1,

but any marker with a similar localization would suffice.

-Figure 5. As for Fig. 2A, also in 5A-B having a marker that stains the nuclear envelope

would help (especially in 5B). The anti-MAD-2 still detects foci surrounding both the

chromatin and the NPCs and therefore, to support their claims the authors should

provide more convincing staining examples.

I would not define as “dramatic” the difference in zone 2 (Fig. 5D, line 304) and I also

do not think it is appropriate to use the term “delayed” here. In fact, a “delayed”

synapsis would imply that at some point the mad-1(A) reached full synapsis, which

instead it never happens. Therefore, it should simply be stated in here that mad-1(A)

displays a defect in SC assembly.

Moreover, by looking at the examples provided in Fig. 5E, the unsynapsed regions

seem to be very small and the authors should make sure that, if my impression is

correct, this is clearly specified in the text. Considering the images, to me it looks like

mad-1(A) is largely proficient in establishing the SC globally, and that only small

portions of chromosomes remain unsynapsed. If instead this phenotype is more

severe than it looks from the pictures, then it should be reflected in the presence of

achiasmatic chromosomes in the diakinesis nuclei: have the authors looked at this? If

not, they should.

Furthermore, since they both have a checkpoint activation, I would

like to see whether the expression/localization of PCH-2 is altered in mad-1(A) and

DN-mad-1.

-Figure 6. Line 353: I suppose the authors used the mad-2(V193N) that has been

already characterized elsewhere? If this is the case, please include the appropriate

reference (Lara-Gonzalez et al.; 2021).

Lines 356-359: this whole section is a bit over-emphasized, since this mutant was

previously characterized, and it was already shown that this “locked-open” version of

MAD-2 is not detectable in the nucleus (Lara-Gonzalez et al.; 2021). Therefore it´s not

surprising that this holds true also in the germ cells.

Lines 365-371: mdf-2 is sitting at the genetic position -6.82 on Ch. IV and spo-11 is at

+4.89, meaning that they are almost 12 cM away from each other: this to me does not

really qualify as “closely linked”. The authors should build the double with spo-11 and

re-assess apoptosis to make sure that it is comparable to the cep-1 data.

Most importantly, given the robust activation of the DNA damage checkpoint in the

mad-2-open mutants, the removal of SPO-11 will univocally show, if that´s the case,

that the persistent damage due to the extensive asynapsis is what triggers the

activation of the checkpoint. The authors do contemplate this possibility in the text,

however it should be directly proved.

It would be nice to see a PCH-2 staining in the mad-2-open mutants as well.

The extent of synapsis defect in the nuclei of the mad-2-open seem to be much more

pronounced compared to the mad-1(A): the authors should perform the analysis of

DAPI-bodies in the diakinesis nuclei of the mad-2-open mutants.

The fact that in the double with cep-1 there is already a full abrogation of the increase

in the apoptosis levels, suggests that the major trigger for the checkpoint activation

involves DNA damage, however I would also like to see if removing pch-2 from the

mad-2-open mutant would make any difference.

As for any control mechanism, I would expect that if in a certain mutant background

there is a substantial activation of a checkpoint, then suppressing it, should elicit

formation of aberrant gametes.

For this reason, DAPI-bodies analysis in the diakinesis nuclei should be conducted

also in the mad-2-open; cep-1 doubles, as it would be interesting to assess which is

the biological significance of preventing activation of these checkpoints during meiotic

prophase I.

-Last but not least: I am aware that mad-1 and mad-2 have been used to refer to mdf-

1/2 for simplicity, but I also believe that is important to stick with the nomenclature in

order to avoid confusion. The fact that this has been done in a couple of different

studies does not necessarily mean that is appropriate. Since mdf-1/2 is the assigned

name to these genes, either the authors request a name change to Wormbase or they

should keep their correct identification names.

Reviewer #2: In C. elegans, synapsis and the synapsis checkpoint depend on cis-acting chromosome regions known as pairing centers. The Bhalla group has previously shown that spindle assembly checkpoint proteins, MAD-1, MAD-2, and BUB-3, regulate and monitor synapsis in a manner that depends on pairing centers (Bohr et al., 2015). In this manuscript, the authors further investigate how Mad1 and Mad2 control these processes. They showed that the processive movement of pairing centers is less frequent in animals lacking MAD-1 or BUB-3, although the average patch size of pairing centers and run length are not affected. Using an N-terminal deletion allele of MAD-1, which disrupts its interaction with Tpr, the authors showed that the localization of MAD1 to the nuclear envelope is required for synapsis checkpoint, but not for synapsis itself. A surprise came from the observation that MAD-2 is localized normally to the nuclear envelope in this MAD-1 mutant, which contrasts with the complete loss of MAD-2 in mad-1 null mutants. Next, the authors demonstrated that MAD-2 does not localize to the nuclear periphery in mad-1 mutants that cannot bind MAD-2, and this results in defects of both the synapsis checkpoint and synapsis. Lastly, the authors explored MAD-2's protein conformation and its effects on the synapsis checkpoint and synapsis. By using an allele that locks MAD-2 in its open conformation, they demonstrated that the closed conformation of MAD-2 is required for synapsis and synapsis checkpoint.

This paper is well written, and the rationale for each experiment is also well justified. The main conclusion of this paper is based on the Mad1-independent localization of Mad2 to the nuclear periphery, which was shown using the N-terminal deletion allele of mad-1. This result is very puzzling as it contradicts the behavior of Mad2 in all the other mad-1 alleles and thus demands a more thorough analysis to validate this finding. The current Figures show just one representative nucleus of each mutant without quantification. I would suggest that the authors show zoomed-out views capturing multiple germline nuclei and also quantify the fluorescence intensity at the nuclear periphery using MAD-2::GFP, like the analysis performed in Figure S3H of Lara-Gonzalez et al., 2019.

Some of the details in the Discussion regarding the localization and function of Mad2 need further clarification. It appears that MAD-2 is absent in mad-1(A) mutants, and the authors speculated that MAD-1 might be involved in shuttling MAD-2 into the meiotic nuclei (line 430). Is MAD-2 found in the cytoplasm within the germline? Can the authors clarify what they mean by “a gain of function” (lines 439-441) when MAD-2 is not present in the nucleus in mad-1(A) mutants? Is there evidence showing that MAD-2 is upregulated in mad-1(A) mutants as suggested in line 440?

Is MAD-2 protein present in mad-2 open mutants? Can the MAD-2 antibody recognize the open form? Can the authors explain the synapsis defects in these mutants as opposed to the accelerated synapsis in mad-2 null?

Here are other points:

1. The first paragraph of the Results section is probably better suited for the Introduction. This background information can be streamlined with the existing Introduction.

2. Regarding the results shown in Figure 1, it is unclear how the reduced frequency of PCM can explain the accelerated synapsis in mad-1 and bub-3 mutants. Isn’t the PCM required for robust synapsis?

3. In lines 191-193, the synaptonemal complex has already been defined in the Introduction (lines 66-68).

4. In lines 205-207, please change the verbs to past tense.

5. After line 242, adding a sentence summarizing the results will be helpful.

6. Is BUB-1 or BUB-3 localization known in the germline? This knowledge will help interpret the results using MAD-1(AAA) and the role of Bub3 in synapsis checkpoint.

7. For the experiments shown in Figure 6B using cep-1, please mention it was previously shown that the synapsis checkpoint is independent of CEP-1 (Bhalla and Dernburg, 2005), as it may not be apparent to readers who are outside of this immediate field.

8. For Figure S1, I would suggest switching the order between MAD-1(A) and MAD-1(AAA), following the order of experiments described in the main text.

Reviewer #3: Devigne and Bhalla explore the interactions of MAD-1 and MAD-2 and their role in regulating synapsis and the synapsis checkpoint. This work expands on previous work from Bohr and Bhalla, which showed that spindle checkpoint components Mad-1, Mad-2, and Bub-3 negatively regulate synapsis and promote the synapsis checkpoint response. First, they find that mad-1 and bub-3 checkpoint mutants have a reduced frequency of PCMs, which is consistent with faster synapsis. Next, they analyze several mad-1 mutants and determine whether MAD-1 and MAD-2 localizes to the nuclear periphery, affect synapsis, and affect the synapsis checkpoint.

Overall, there are some intriguing data with the decrease in PCMs and the analysis of the different mutants, but the story seemed incomplete.

1) The data in Figure 1 was not tied well together with the mutant analysis. It read as tying up loose ends from a previous manuscript without a strong rationale for how it fits with the other data in the manuscript.

2) In Figure 2A, it does look like there is some co-localization in the deltaN-mad-1 mutant. Can the authors perform a quantitative analysis on the percent co-localization in wildtype and mutant?

3) In Figure 2C, the delta-N-mad-1; syp-1 double mutant has more apoptosis than the spo-11; syp-1 double mutant. Is this difference significant? If so, doesn’t the result suggest that the DNA damage checkpoint is also being triggered in the delta-N-mad-1; syp-1 double?

4) Why is Figure S3 a supplemental figure. It seems important and should be part of Figure 6.

5) What epitope does the MAD-2 antibody recognize and can this account for why a mad-2-open mutant version does not show MAD-2 staining?

6) Do the mad-1 mutants have defects in PCMs (for example, the delta-N-mad-1 and the mad-1-A)? What about the mad-2-open mutant?

7) The story ended without a strong understanding of why the different mutants gave the different phenotypes and how the mutant analysis could be tied together in a model for how MAD-1 and MAD-2 function in synapsis and the synapsis checkpoint. Can the authors provide a more comprehensive model?

**********

**Have all data underlying the figures and results presented in the manuscript been provided?**

Large-scale datasets should be made available via a public repository as described in the *PLOS Genetics* data availability policy, and numerical data that underlies graphs or summary statistics should be provided in spreadsheet form as supporting information.

Reviewer #1: Yes

Reviewer #2: Yes

Reviewer #3: Yes

**********

PLOS authors have the option to publish the peer review history of their article (what does this mean?). If published, this will include your full peer review and any attached files.

If you choose “no”, your identity will remain anonymous but your review may still be made public.

**Do you want your identity to be public for this peer review?** For information about this choice, including consent withdrawal, please see our Privacy Policy.

Reviewer #1: No

Reviewer #2: No

Reviewer #3: No

# Author comment: Author response to Decision Letter 0

**DOI:** 10.1371/journal.pgen.1009598.r002

29 Sep 2021

Attachment Submitted filename: coverletterDevigneandBhalla.pdf Click here for additional data file.

# Peer review: Decision Letter 1

**DOI:** 10.1371/journal.pgen.1009598.r003

**Authors:** Gregory P. Copenhaver, JoAnne Engebrecht

11 Oct 2021

Dear Needhi,

We are pleased to inform you that your manuscript entitled "Mad1’s ability to interact with Mad2 is essential to regulate and monitor meiotic synapsis in C. elegans" has been editorially accepted for publication in PLOS Genetics. Congratulations!

Thank you for thoroughly addressing the previous reviews. Reviewers 1 and 2 have suggested minor text changes to improve clarity that I ask you to consider. I would also ask you to consider changing "wild-type levels of apoptosis" (e.g., lines 137, 257, 348, perhaps other places) in the various mutants to somehow indicate that checkpoint-dependent apoptosis is impaired and apoptosis is at un-induced levels (which is not wild type in the mutant condition). You can attend to these minor issues as you prepare your final draft for the production team (the editorial team will not need to re-evaluate).

Before your submission can be formally accepted and sent to production you will need to complete our formatting changes, which you will receive in a follow up email. Please be aware that it may take several days for you to receive this email; during this time no action is required by you. Please note: the accept date on your published article will reflect the date of this provisional acceptance, but your manuscript will not be scheduled for publication until the required changes have been made.

Once your paper is formally accepted, an uncorrected proof of your manuscript will be published online ahead of the final version, unless you’ve already opted out via the online submission form. If, for any reason, you do not want an earlier version of your manuscript published online or are unsure if you have already indicated as such, please let the journal staff know immediately at plosgenetics@plos.org.

In the meantime, please log into Editorial Manager at https://www.editorialmanager.com/pgenetics/, click the "Update My Information" link at the top of the page, and update your user information to ensure an efficient production and billing process. Note that PLOS requires an ORCID iD for all corresponding authors. Therefore, please ensure that you have an ORCID iD and that it is validated in Editorial Manager. To do this, go to ‘Update my Information’ (in the upper left-hand corner of the main menu), and click on the Fetch/Validate link next to the ORCID field.  This will take you to the ORCID site and allow you to create a new iD or authenticate a pre-existing iD in Editorial Manager.

If you have a press-related query, or would like to know about making your underlying data available (as you will be aware, this is required for publication), please see the end of this email. If your institution or institutions have a press office, please notify them about your upcoming article at this point, to enable them to help maximise its impact. Inform journal staff as soon as possible if you are preparing a press release for your article and need a publication date.

Thank you again for supporting open-access publishing; we are looking forward to publishing your work in PLOS Genetics!

Yours sincerely,

JoAnne Engebrecht

Guest Editor

PLOS Genetics

Gregory P. Copenhaver

Editor-in-Chief

PLOS Genetics

www.plosgenetics.org

Twitter: @PLOSGenetics

----------------------------------------------------

Comments from the reviewers (if applicable):

Reviewer's Responses to Questions

**Comments to the Authors:**

**Please note here if the review is uploaded as an attachment.**

Reviewer #1: In the revised version of their manuscript, the authors have provided further experimental evidence that supports their claims. They addressed my concerns both experimentally and by modifying the text. I found a few minor things that should be changed but overall, I have no further issues with this manuscript and therefore I support publication in PLoS Genetics.

Line 165: it should be “…remains on chromosomes until early….”;

Line 175: there is no synapsis shown in Fig 2D, the authors should double-check the figure call;

Line 371: “when we generate monitor DAPI….” this sentence does not make sense, I suppose the genotype mdf-2mad-2-open; cep-1 should go in there?

Line 541: “Antibodies against SUN-1….” the source of this reagent should also be acknowledged as for the others.

Reviewer #2: The authors addressed all the reviewers’ concerns and significantly improved the manuscript.

Here are some minor comments:

1. In lines 35 and 99, it is a bit of an overstatement to say that Mad2 is a “major” regulator of meiotic synapsis. Mad2 mutants exhibit modest synapsis defects, but majority of oocytes ultimately can support crossover formation.

2. In line 80, it would be helpful for general audience to briefly introduce what the spindle assembly checkpoint is and how it functions.

3. References are needed in lines 92, 141, and 203.

4. In line 268, although it might be statistically significant, 5.7% is only a minor fraction.

5. In line 398, please add “in” before “mdf-2 (mad-2) open mutants”.

6. In line 454, please change “monitors” to “monitor”.

7. Please add the strain information mdf-1::gfp (Figure 1A) (or was it gfp::mdf-1?) to the worm strain list.

Reviewer #3: The authors have responded to all of my concerns with either new analysis or rewriting. The story is now stronger, showing that the interaction between Mad1 and Mad2 is important for monitoring synapsis. Especially interesting is that the conformation change of Mad2 is needed for the synapsis checkpoint. This paper will be important for the meiosis and spindle checkpoint fields.

**********

**Have all data underlying the figures and results presented in the manuscript been provided?**

Large-scale datasets should be made available via a public repository as described in the *PLOS Genetics* data availability policy, and numerical data that underlies graphs or summary statistics should be provided in spreadsheet form as supporting information.

Reviewer #1: Yes

Reviewer #2: Yes

Reviewer #3: Yes

**********

PLOS authors have the option to publish the peer review history of their article (what does this mean?). If published, this will include your full peer review and any attached files.

If you choose “no”, your identity will remain anonymous but your review may still be made public.

**Do you want your identity to be public for this peer review?** For information about this choice, including consent withdrawal, please see our Privacy Policy.

Reviewer #1: No

Reviewer #2: No

Reviewer #3: No

----------------------------------------------------

**Data Deposition**

If you have submitted a Research Article or Front Matter that has associated data that are not suitable for deposition in a subject-specific public repository (such as GenBank or ArrayExpress), one way to make that data available is to deposit it in the Dryad Digital Repository. As you may recall, we ask all authors to agree to make data available; this is one way to achieve that. A full list of recommended repositories can be found on our website.

The following link will take you to the Dryad record for your article, so you won't have to re‐enter its bibliographic information, and can upload your files directly: 

http://datadryad.org/submit?journalID=pgenetics&manu=PGENETICS-D-21-00626R1

More information about depositing data in Dryad is available at http://www.datadryad.org/depositing. If you experience any difficulties in submitting your data, please contact help@datadryad.org for support.

Additionally, please be aware that our data availability policy requires that all numerical data underlying display items are included with the submission, and you will need to provide this before we can formally accept your manuscript, if not already present.

----------------------------------------------------

**Press Queries**

If you or your institution will be preparing press materials for this manuscript, or if you need to know your paper's publication date for media purposes, please inform the journal staff as soon as possible so that your submission can be scheduled accordingly. Your manuscript will remain under a strict press embargo until the publication date and time. This means an early version of your manuscript will not be published ahead of your final version. PLOS Genetics may also choose to issue a press release for your article. If there's anything the journal should know or you'd like more information, please get in touch via plosgenetics@plos.org.

# Editor report: Acceptance letter

**DOI:** 10.1371/journal.pgen.1009598.r004

**Authors:** Gregory P. Copenhaver, JoAnne Engebrecht

1 Nov 2021

PGENETICS-D-21-00626R1 

Mad1’s ability to interact with Mad2 is essential to regulate and monitor meiotic synapsis in C. elegans 

Dear Dr Bhalla, 

We are pleased to inform you that your manuscript entitled "Mad1’s ability to interact with Mad2 is essential to regulate and monitor meiotic synapsis in C. elegans" has been formally accepted for publication in PLOS Genetics! Your manuscript is now with our production department and you will be notified of the publication date in due course.

The corresponding author will soon be receiving a typeset proof for review, to ensure errors have not been introduced during production. Please review the PDF proof of your manuscript carefully, as this is the last chance to correct any errors. Please note that major changes, or those which affect the scientific understanding of the work, will likely cause delays to the publication date of your manuscript. 

Soon after your final files are uploaded, unless you have opted out or your manuscript is a front-matter piece, the early version of your manuscript will be published online. The date of the early version will be your article's publication date. The final article will be published to the same URL, and all versions of the paper will be accessible to readers.

Thank you again for supporting PLOS Genetics and open-access publishing. We are looking forward to publishing your work! 

With kind regards,

Andrea Szabo

PLOS Genetics

On behalf of:

The PLOS Genetics Team

Carlyle House, Carlyle Road, Cambridge CB4 3DN | United Kingdom

plosgenetics@plos.org | +44 (0) 1223-442823

plosgenetics.org | Twitter: @PLOSGenetics
